# A positive mechanobiological feedback loop controls bistable switching of cardiac fibroblast phenotype

**DOI:** 10.1038/s41421-022-00427-w

**Published:** 2022-09-06

**Authors:** Lele Niu, Bo Cheng, Guoyou Huang, Kai Nan, Shuang Han, Hui Ren, Na Liu, Yan Li, Guy M. Genin, Feng Xu

**Affiliations:** 1 The Key Laboratory of Biomedical Information Engineering of Ministry of Education, School of Life Science and Technology, Xi’an Jiaotong University, Xi’an, Shaanxi China; 2 Bioinspired Engineering and Biomechanics Center (BEBC), Xi’an Jiaotong University, Xi’an, Shaanxi China; 3 Department of Engineering Mechanics, School of Civil Engineering, Wuhan University, Wuhan, Hubei China; 4 Department of Orthopedics Surgery, The First Affiliated Hospital of Xi’an Jiaotong University, Xi’an, Shaanxi China; 5 Honghui Hospital, Xi’an, Shaanxi China; 6 Department of Respiratory and Critical Care Medicine, The First Affiliated Hospital of Xi’an Jiaotong University, Xi’an, Shaanxi China; 7 Department of Gastroenterology, The Second Affiliated Hospital of Xi’an Jiaotong University, Xi’an, Shaanxi China; 8 Department of Cardiology, Tangdu Hospital, Fourth Military Medical University, Xi’an, Shaanxi China; 9 Department of Mechanical Engineering & Materials Science, Washington University in St. Louis, St. Louis, MO USA; 10 NSF Science and Technology Center for Engineering Mechanobiology, Washington University in St. Louis, St. Louis, MO USA

**Keywords:** Mechanotransduction, Mechanisms of disease

## Abstract

Cardiac fibrosis is associated with activation of cardiac fibroblasts (CFs), a pathological, phenotypic transition that is widely believed to be irreversible in the late stages of disease development. Sensing of a stiffened mechanical environment through regulation of integrin-based adhesion plaques and activation of the Piezo1 mechanosensitive ion channel is known to factor into this transition. Here, using integrated in vitro and in silico models, we discovered a mutually reinforcing, mechanical positive feedback loop between integrin β1 and Piezo1 activation that forms a bistable switch. The bistable switch is initiated by perturbations in matrix elastic modulus that amplify to trigger downstream signaling involving Ca^2+^ and YAP that, recursively, leads fibroblasts to further stiffen their environment. By simultaneously interfering with the newly identified mechanical positive feedback loop and modulating matrix elastic modulus, we reversed markers of phenotypical transition of CF, suggesting new therapeutic targets for fibrotic disease.

## Introduction

Bistable switches arising from cascades of mutually reinforcing or inhibitory chemical reactions enable a range of decision-making in living cells^1^. Such switches can enact stable or possibly irreversible changes to cell physiology via series of reversible biochemical reactions. These include many of the reactions involved in cell adhesion, spreading, and differentiation^2,3^ such as protein phosphorylation and dephosphorylation^4,5^, receptor–ligand binding and unbinding^6^, and protein folding and unfolding^7,8^. Irreversible changes to cell physiology are important to physiological development^9–11^, but also underlie pathologies such as fibrosis^12^. For instance, cardiac fibrosis involves an irreversible phenotypic transition of cardiac fibroblasts (CFs) to a chronically activated state^13^, rendering the disease incurable. The phenotypic transition from fibroblast to myofibroblast can be triggered by changes in matrix elastic modulus, indicating that mechanics interacts with biochemical reactions in this case.

Many instances of bistable switching arise from positive feedback loops (PFLs) in which reactions whose products activate one other create a hypersensitive system with output that switches rapidly to a high and stable value when a stimulus exceeds a critical threshold^14–16^. PFLs thereby enable bistable switching in response to an external stimulus^14,15,17^ in a range of biochemically triggered, irreversible cell behaviors^18^ including MAPK-mediated oocyte maturation^14,15^ and Twist1-Prrx1-TNC-mediated fibroblast activation^19^. We therefore hypothesized that mechanosensing associated with cardiac fibrosis^20,21^ may trigger a PFL that leads to irreversible phenotypic switching.

We studied two candidate mechanosensitive membrane proteins as potential participants in this PFL, i.e., the adhesion protein integrin and the ion channel Piezo1^22–24^. Integrins and their downstream signaling on the FAK-YAP signaling axis are mechanosensitive mediators of CF activation in response to mechanosensing of stiff tissues^25,26^. The Piezo1 channel^27^, a channel widely expressed in myocardial tissue converts mechanical stimuli to Ca^2+^ signaling^28–31^. The two sets of proteins interact, with activated Piezo1 upregulating assembly of focal adhesions and activating the integrin-FAK signal pathway in certain tumors and in aortic valve stenosis^32,33^. We therefore investigated whether integrins could also regulate Piezo1, thereby forming a PFL that acts as a bistable switch for the CF phenotypic transition associated with irreversible cardiac fibrosis.

After identifying a relationship between tissue stiffening and upregulation of Piezo1 and integrin β1 in a rat model of myocardial infarction (MI), we used integrated in vitro hydrogel culture and in silico mathematical models to explore the potential for a mechanical PFL between Piezo1 and integrin β1 in stiffness-induced CF phenotypic transition. This led to the discovery of a mechanical PFL that locks CFs into the well-known active state that persists even in the absence of high elastic modulus of ECM and is seemingly irreversible. However, we then discovered that simultaneously interfering with this newly identified mechanical PFL and reducing matrix elastic modulus enabled a degree of reversibility of CF activation with potential therapeutic applications.

## Results

### Expressions of Piezo1 and integrin β1 increase with tissue stiffening during development of cardiac fibrosis

**Fig. 1:**
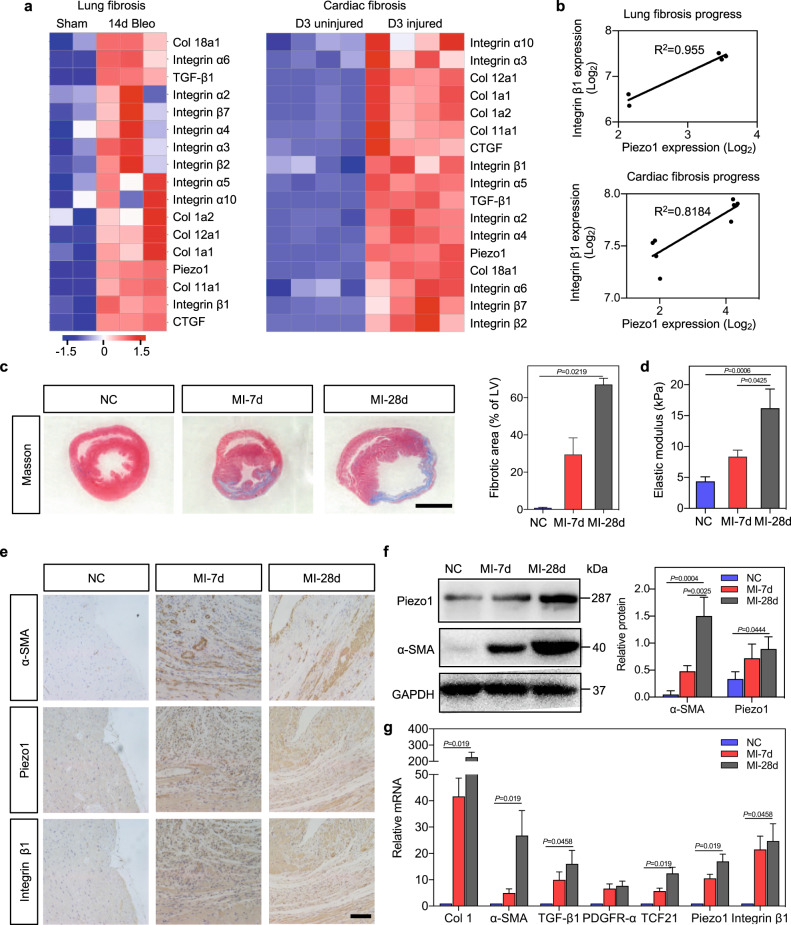
Enhanced expression of Piezo1 and integrin β1 arising from tissue stiffening during the onset of cardiac fibrosis. **a** Meta-analysis of published GEO data analysis showing Piezo1, integrin, collagen and connective tissue growth factor (CTGF) expression in lung fibrosis (bleomycin-mediated lung fibrosis, “Bleo”) and heart with sham and injury. **b** Correlation of Piezo1 and integrin β1 expression in the lung and heart with sham and fibrosis in GEO data. **c** Representative images of Masson’s trichrome-stained sections from the negative control (NC) and MI rats. Scale bar, 5 mm (LV: left ventricular). **d** Elastic modulus of the heart sections from NC and MI rats. The elastic modulus of the heart tissue increases from 4.35 kPa (NC) to 8.38 kPa (MI-7d) finally to 16.22 kPa (MI-28d). **e** Immunohistochemical staining of α-SMA, integrin β1, and Piezo1 in the cardiac tissues of NC and MI rats. Scale bar, 200 μm. **f** Relative protein levels of α-SMA and Piezo1 determined by Western blot analysis. **g** RT-PCR analysis of Col 1, α-SMA, TGF-β1, PDGFR-α, TCF21, Piezo1, and integrin β1 in NC and MI rats.

Analysis of the expression of integrin β1 and Piezo1 in the Gene Expression Omnibus (GEO; accession numbers GSE161322, GSE152250, and GSE151834) revealed both to be highly expressed across a range of fibrotic tissues (Fig. 1a and Supplementary Fig. S1a), with expression highly correlated during development of fibrotic disease (Fig. 1b and Supplementary Fig. S1b). We hypothesized that these coordinated increases in expression correlate with tissue stiffening and tested this hypothesis by studying the development of cardiac fibrosis in a rat model of post-infarction cardiac fibrosis.

Following the induction of myocardial infarction (MI) in our rat model, a fibrotic accumulation of collagen developed over time (Fig. 1c), while the wall thickness of the infarcted myocardium decreased (Supplementary Fig. S2a, b) and the effective elastic modulus of myocardial tissue increased from 4.35 ± 0.76 kPa (negative controls, NC) to 8.38 ± 1.04 kPa (7 days post-infarct, MI-7d) to 16.21 ± 3.09 kPa (28 days post-infarct, MI-28d) (Fig. 1d). Studies have shown that Piezo1 and β1 subunit of integrins play key roles during the development of cardiac fibrosis^3,29,30,34^. Consistently, expressions of Piezo1 and integrin β1 were significantly higher in MI tissue than in tissue from NC rats (*p* < 0.01), and correlated over time with both the elastic modulus of the myocardium (*R*^2^ > 0.65), with expression of the activated myofibroblast protein marker α-smooth muscle actin (α-SMA) (*R*^2^ > 0.52) and with fibrosis genetic markers Col1, TGF-β1, TCF21, and PDGFR-α (*R*^2^ > 0.52) (Fig. 1e–g and Supplementary Fig. S3). Expressions of Piezo1 and integrin β1 in vivo thus correlated with each other, with tissue stiffening, and with the development of cardiac fibrosis.

### Piezo1 and integrin β1 expressions and CF activation correlate with elastic modulus of matrix in vitro

**Fig. 2:**
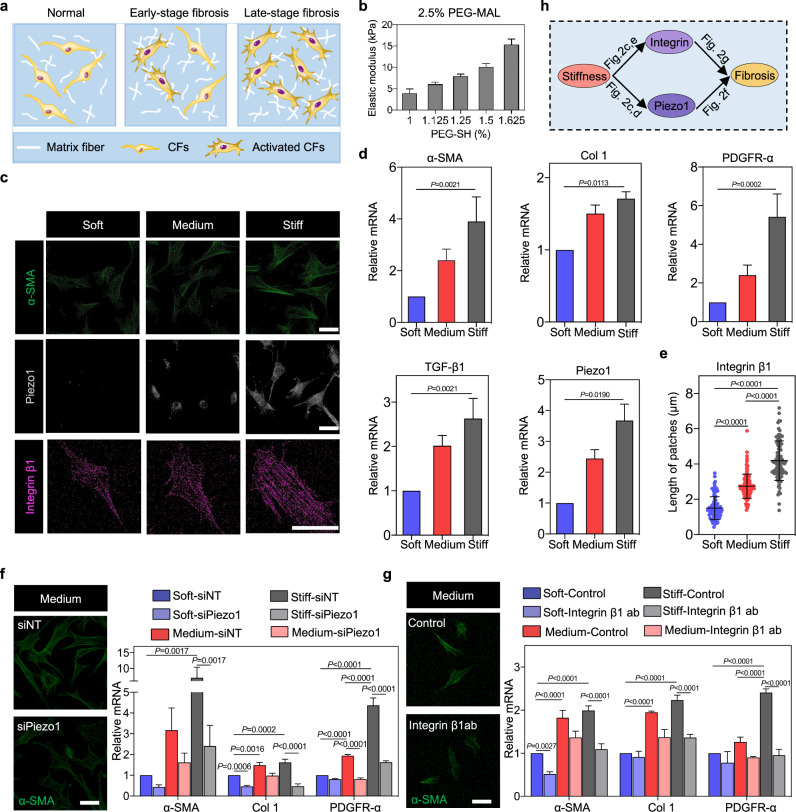
Matrix stiffness-mediated increases of Piezo1 and integrin β1 promote CF activation in vitro. **a** Schematic of different stages of cardiac fibrosis. In injured regions, accumulation of ECM proteins promotes tissue stiffening. **b** Moduli of peptide-modified PEG-MAL hydrogels with increasing concentration of PEG-SH. PEG-SH was swollen into the network (2.5% of PEG-MAL) at 1%, 1.125%, 1.25%, 1.5%, and 1.625%, resulting in hydrogels with stiffness of 4, 6, 8, 9, and 15 kPa, respectively. Elastic modulus was measured by rotational rheometry. **c** Immunofluorescence analysis indicated enhanced activation of CFs with increasing matrix stiffness (gray, Piezo1; green, α-SMA; Pink, Integrin β1). Scale bar, 50 μm. **d** RT-PCR analysis of α-SMA, PDGFR-α, TGF-β1 and Col 1 in CFs after 7-day culture. **e** Quantifications of integrin β1 plaque size on matrices of different elastic modulus (*n* ≥ 90 data points). **f** Immunofluorescence and RT-PCR analyses of α-SMA, Col 1 and PDGFR-α indicated that activation of CFs decreased following siRNA silencing of Piezo1 (green, α-SMA). Scale bar, 50 μm. **g** Immunofluorescence and RT-PCR analyses of α-SMA, Col 1 and PDGFR-α indicated that activation of CFs decreased in integrin β1-depleted CFs, both with and without integrin-blocking antibodies (green, α-SMA). Scale bar, 50 μm.

To validate this relationship between ECM stiffening, expressions of Piezo1 and integrin β1, and CF activation in vitro, rat CFs were cultured on PEG hydrogel matrices with elastic modulus of 4 kPa (“soft”, representative of NC), 8 kPa (“medium”, representative of MI-7d) and 15 kPa (“stiff”, representative of MI-28d) (Fig. 2a, b and Supplementary Fig. S4). Immunofluorescence staining and real-time PCR (RT-PCR) revealed that expression of α-SMA, a marker of CF activation, upregulated with increasing matrix elastic modulus (Fig. 2c, d and Supplementary Fig. S5), consistent with previous studies^35^. Expressions of CF activation markers Col 1, PDGFR-α, and TGF-β1 upregulated with increasing matrix elastic modulus (Fig. 2d), as did expressions of Piezo1 and integrin β1 (Fig. 2c–e and Supplementary Fig. S5). Thus, consistent with effects observed in cardiac fibrosis in vivo, matrix stiffening in vitro correlated with CF activation and with increased Piezo1 and integrin β1 expressions.

To establish causation, we performed small interfering RNA (siRNA) silencing of Piezo1 in CFs seeded on matrices of different elastic modulus. The knockdown efficiency of Piezo1 was confirmed by measuring mRNA expression levels (Supplementary Fig. S6). Piezo1-depleted CFs showed lower expressions of α-SMA, Col 1 and PDGFR-α than did control CFs treated with negative siRNA (Fig. 2f and Supplementary Fig. S7), as well as reduced proliferation (Supplementary Fig. S8). Next, we used an antibody to block integrin β1-mediated signaling and confirmed that integrin β1 was indeed inhibited at protein levels (Supplementary Fig. S9). Similar to Piezo1-depleted CFs, integrin β1-depleted CFs showed decreased expressions of α-SMA and other activation markers (Col 1 and PDGFR-α) compared to control CFs (Fig. 2g and Supplementary Fig. S10). Meanwhile, we confirmed our main results by using siRNA silencing of integrin β1 which showed that integrin β1 can indeed promote activation of CFs and enhance the level of Piezo1 (Supplementary Figs. S11, S12). Collectively, these results indicate that increased expressions of Piezo1 and integrin β1 expression are required for CF to activate in response to increasing matrix elastic modulus in vitro.

### Stiffness-mediated PFL drives cardiac fibroblast activation

**Fig. 3:**
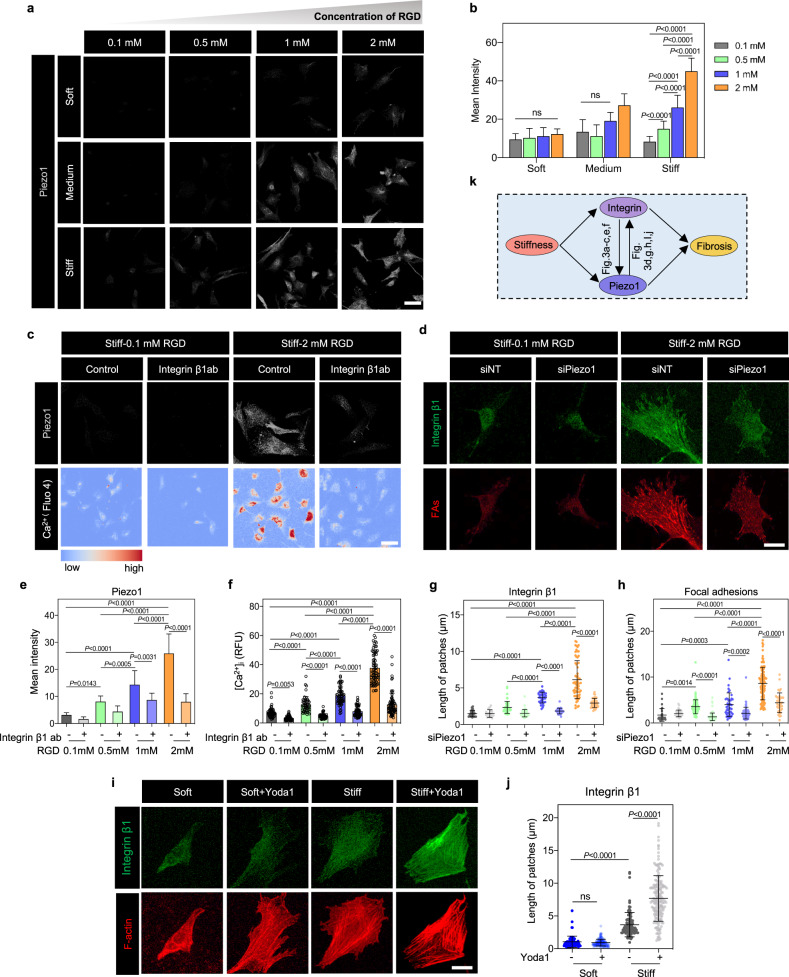
A stiffness-triggered positive feedback loop between Piezo1 and integrin β1 emerges during CF activation. **a** Immunofluorescence analysis indicated that, in CFs cultured on stiff matrices, but not in CFs cultured on “medium” or “soft” matrices, Piezo1 expression increased with increasing RGD concentration on the matrix (grey, Piezo1). Scale bar, 50 μm. **b** Quantification of the immunofluorescence staining in panel **a** (*n* ≥ 17 cells). **c** Blocking of integrin β1 by antibodies eliminated these effects in CFs cultured on stiff matrices, reducing both expression of Piezo1 (grey, Piezo1) and cytosolic Ca^2+^ concentrations (Fluo-4 AM). Scale bar, 50 μm. **d** Knockdown of Piezo1 with siPiezo1 reduced expression of integrin β1 (green) and the focal adhesion proteins vinculin and paxillin (red). Scale bar, 10 μm. **e**, **f** Quantification of the fluorescence intensities in panel **c** (**e** *n* ≥ 11 cells; **f** *n* ≥ 50 cells). **g**, **h** Quantifications of the data in panel **d** (*n* ≥ 17 data points). **i** Overexpression of Piezo1 with Yoda1 increased expression of integrin β1 on stiff hydrogels but not soft matrix. Scale bar, 10 μm. **j** Quantifications of the data in panel **i** (*n* ≥ 75 data points). **k** Schematic diagram of the PFL.

To test our hypothesis that the mechanosensitive elevations of integrin β1 and Piezo1 expression observed over the development of cardiac fibrosis serve to promote CF activation through a PFL-mediated bistable switch, we determined whether the mathematical criterion for a bistable switch was met, namely that mutual inhibition or promotion occurred. We therefore first established that integrin β1 promoted expression of Piezo1 on PEG hydrogel matrices of the three elastic moduli, each with prescribed concentrations of RGD, a ligand that binds to and increases the activation of integrin β1^36,37^. Piezo1 expression did indeed increase with the RGD ligand concentration, but only for CFs cultured on the stiff matrix (Fig. 3a, b). This was the case even when the increased cell spreading area associated with increased RGD concentration was considered (Supplementary Fig. S13a, b). Cytosolic Ca^2+^, an indicator of Piezo1 activity^38–41^, also increased in CFs cultured on stiff matrices with a higher RGD concentration (Supplementary Fig. S14a, b). We verified these results by blocking integrin β1 (Fig. 3c, e, f and Supplementary Fig. S15a), which reduced both Piezo1 expression and cytosolic Ca^2+^ concentrations. Thus, Piezo1 expression and activity were upregulated by integrin β1 activation only in CFs cultured on stiff matrices, meaning that an integrin β1-Piezo1-mediated PFL would be possible only on these “stiff” matrices.

To test the other arm of the PFL on stiff matrices, we verified that Piezo1 also promotes integrin β1 by culturing Piezo1-depleted CFs on stiff matrices. Fewer focal adhesions formed, as characterized by concentrations of paxillin and vinculin at the cell periphery (Fig. 3d, g, h and Supplementary Fig. S15b), indicating that Piezo1 promotes the activity of integrin at focal adhesions. To further assess the stiffness-dependent activation of integrin β1 by Piezo1, we overexpressed Piezo1 through treatment of Yoda1. Increased Piezo1 can indeed increase the expression level of integrin β1 and length of FAs on stiff matrices (but no effects on soft matrices) (Fig. 3i, j). This mutual promotion of activity indicated a potential PFL mediated by Piezo1 and integrin β1 under conditions associated with the development of cardiac fibrosis (Fig. 3k), and motivated the exploration of a role for this PFL in phenotypic switching of cardiac fibroblasts.

### Integrin β1 and Piezo1 upregulate activation synergistically

We next explored the cooperative function of integrin β1 and Piezo1. We examined their joint function by measuring nuclear translocation of Yes-associated protein (YAP) that is associated with integrin-based mechanosensing and concentration of Ca^2+^ that is associated with a host of channels including Piezo1. YAP is involved in CF activation, as evidenced in vivo by increased expressions of YAP and its target genes connective tissue growth factor (CTGF) and cysteine-rich 61 (*CYR61*) during the development of cardiac fibrosis in our rat MI model (Supplementary Fig. S16a, b). This was confirmed in vitro, with knockdown of integrin β1 by antibody treatment decreasing nuclear localization of YAP (measured by the *R*_nc_, the nucleus-to-cytoplasm ratio of YAP) and decreasing activation of the YAP target genes *CTGF* and *CYR61* (Supplementary Fig. S17a–c). Recent studies have also shown that integrin-linked focal adhesion kinase (FAK) can activate YAP signaling, leading to increased α-SMA expression in cardiac fibrosis^42,43^. Taken together, these results confirmed that YAP signaling is downstream of integrin β1 in matrix elastic modulus-regulated CF activation.

**Fig. 4:**
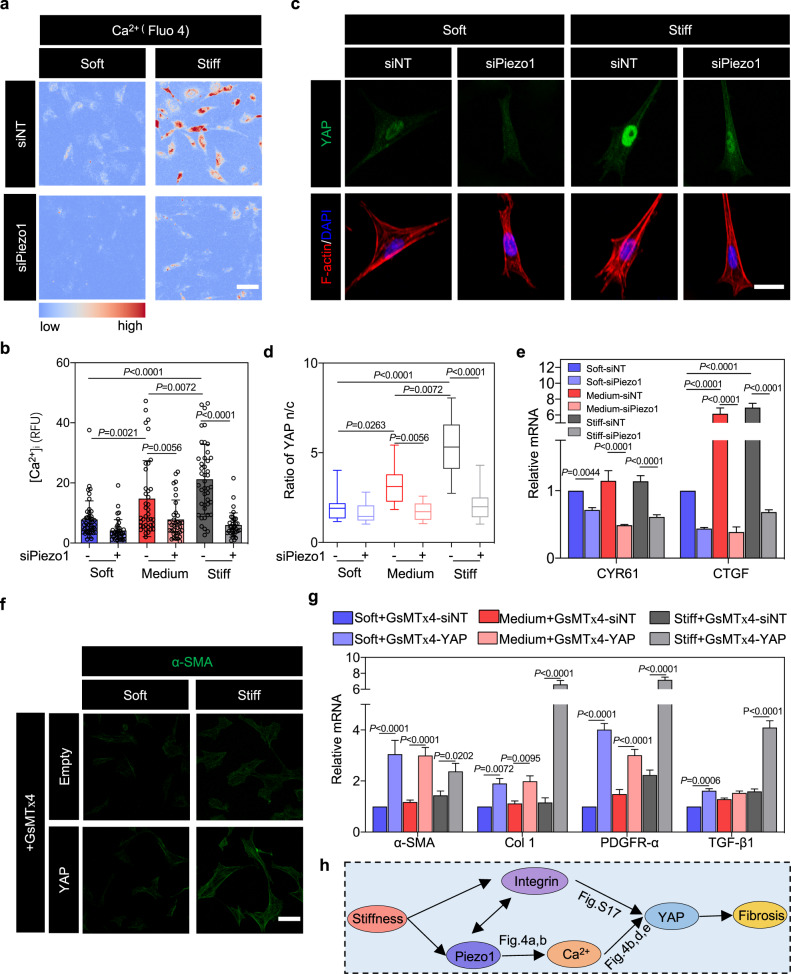
Ca^2+^ and YAP play important roles in integrin β1-Piezo1 PFL-mediated CF activation. **a** Ca^2+^ (Fluo-4 AM) imaging of CFs transfected with siRNA targeting Piezo1 (siPiezo1) or with negative control siRNA (siNT), showing reduction of mechanosensitive cytosolic Ca^2+^ elevation after 3 days of culture in CFs with Piezo1 knocked down. Scale bar, 50 μm. **b** Quantification of fluorescence intensity data from panel **a** (*n* ≥ 33 cells). **c** Immunofluorescence analysis indicated that nuclear YAP decreased following siPiezo1 inhibition of Piezo1 (blue, nucleus (DAPI); green, YAP; red, F-actin). Scale bar, 10 μm. **d** Quantification of the YAP nuclear-to-cytoplasmic ratio from images represented by those in panel **c** (*n* ≥ 12 cells). **e** RT-PCR analysis of downstream YAP genetic targets (*CYR61* and *CTGF*) in CFs with or without Piezo1 knockdown by siPiezo1. **f** Immunofluorescence analysis revealed that α-SMA (green), a marker of CF differentiation, was attenuated by the Piezo1 blocker GsMTx4, but that mechanosensitive α-SMA expression could be rescued by transfecting CFs to produce excess YAP. Scale bar, 50 μm. **g** RT-PCR analysis of α-SMA, Col 1, PDGFR-α and TGF-β1 in YAP-overexpressing CFs in the presence of GsMTx4 indicates that CF activation in response to matrix stiffness is enhanced by Piezo1 and can be rescued by YAP overexpression. **h** Schematic diagram of signaling downstream of the PFL.

Later, we investigated the effects of matrix elastic modulus on Piezo1-mediated activation of Ca^2+^-YAP signaling axis. Results showed that the concentrations of cytosolic Ca^2+^ by Fluo-4 AM imaging were enhanced with increased matrix elastic modulus (Supplementary Fig. S18a, b). Besides, a decrease of cytosolic Ca^2+^ concentration was observed in Piezo1-depleted CFs on matrices with different elastic moduli (soft, medium, and stiff) compared to control cells (Fig. 4a, b and Supplementary Fig. S18c). The mechanism of such observations may be because that stiff matrix can increase membrane tension and thus induce opening of Piezo1^44^, finally resulting in increased cytosolic Ca^2+^. Later, we checked whether Piezo1 is sufficient to activate the cytosolic Ca^2+^ on matrices with different elastic moduli. Application of the Piezo1 agonist (i.e., Yoda1) to CFs cultured on soft matrices can increase the cytosolic Ca^2+^ concentration, while similar application of the spider venom inhibitor of mechanosensitive cation channels GsMTx4 can reduce the cytosolic Ca^2+^ concentration in CFs cultured on stiff matrices (Supplementary Fig. S18d, e). Thus, these results indicated that matrix stiffness can enhance the cytosolic Ca^2+^ concentration via Piezo1 during CF activation.

To investigate the relationships between Piezo1 and YAP during CF activation, knockdown of Piezo1 in CFs was performed. Knockdown of Piezo1 reduces YAP-mechanosensing, with both *R*_nc_ (Fig. 4c, d) and expression of YAP’s transcriptional targets (*CYR61* and *CTGF*) decreasing in CFs on soft, medium and stiff matrices (Fig. 4e). YAP expression was attenuated by GsMTx4, as were markers of CF phenotypic transition including α-SMA, and these markers could be rescued by transfection to overexpress YAP in CFs (Fig. 4f, g and Supplementary Fig. S19a, b), as further confirmed by RT-PCR analysis (Fig. 4g). These results indicated that both integrin β1 and Piezo1 could activate CFs through YAP signaling (Fig. 4h).

### Reversal of CF activation by simultaneously interfering with the integrin β1-Piezo1 PFL combining with reduced elastic modulus of matrix

**Fig. 5:**
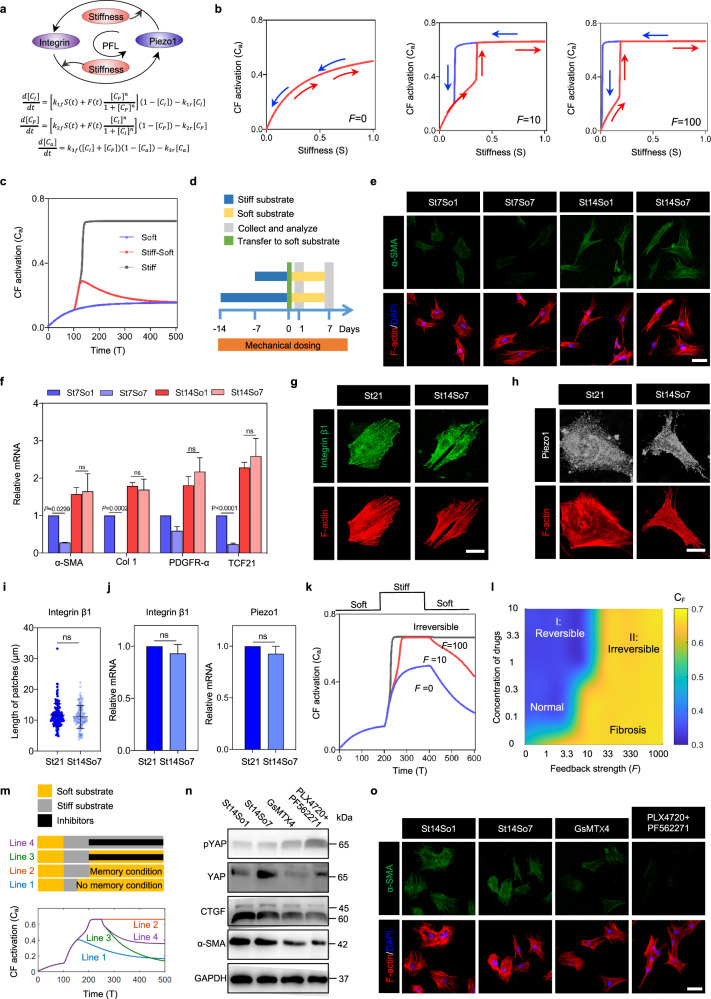
A theoretical model guides conditions under which otherwise irreversible phenotypic transition can be reversed by inhibiting the mechanical PFL and reducing elastic modulus of matrix. **a** Schematic diagram of the matrix stiffness-induced integrin β1-Piezo1 PFL and the mathematical description of the loop (*C*_I_, integrin concentration; *C*_P_, Piezo1 concentration; *C*_a_, CF activation; *S*(*t*), Elastic modulus; *F*(*t*), feedback strength). **b** Modeling results indicated that bifurcation (bistability, that is from reversible state to irreversible state) occurred when *F*[*t*] increased. **c** Modeling results indicated that activation became irreversible following exposure to sufficiently stiff matrices for sufficiently long-time intervals. **d** CFs were cultured on the plate (blue) in growth media for 1–14 days before trypsinization and transfer (light green, day 0) to soft hydrogels (orange). CFs were cultured subsequently on soft hydrogels for 1 and 7 days in growth media before collection and analysis (grey, day 1 and 7). **e** Immunofluorescence analysis of α-SMA in CFs after 1 and 7 days on soft hydrogels with different mechanical doses (blue, nucleus; green, a-SMA; red, F-actin). Scale bar, 50 μm. **f** RT-PCR analysis of α-SMA, Col 1 and PDGFR-α in CFs after 1 or 7 days on soft hydrogels with various mechanical doses. **g**, **h** Immunofluorescence analysis of integrin β1 and Piezo1 in CFs after 7 days on soft hydrogels and 21 days on stiff hydrogels (green, integrin β1; grey, Piezo1; red, F-actin). Scale bar, 10 μm. **i** Quantifications of length of adhesions in panel **g** (*n* ≥ 124 data points). **j** RT-PCR analysis of integrin β1 and Piezo1 in CFs after 7 days on soft hydrogels and 21 days on stiff hydrogels. **k** Our mathematical model predicted that inhibiting feedback strength can reverse activation of CFs. **l** Phase diagram illustrating the irreversibility of cardiac fibrosis with respect to the feedback strength and concentration of PFL inhibitors. **m** CF activation can be reversed, to the greatest extent, by simultaneously inhibiting the mechanical PFL and reducing matrix elastic modulus. **n**, **o** GsMTx4 (Piezo1 inhibitor) and the combination of PLX4720 and PF562271 (inhibiting integrin β1) reduced activation of fibroblasts in conditions for which phenotypic reversal was otherwise impossible (St14), as seen by Western blot and immunofluorescence analysis. Scale bar, 50 μm.

To investigate how an integrin β1-Piezo1-mediated mechanical PFL could be manipulated to affect CF activation, we extended the chemical PFL model of Yeo, et al.^19^, to account for CF mechanosensing and mechanoresponses (Fig. 5a). Our modeling results suggested that bifurcation from a reversible to an irreversible activation state was possible with sufficient feedback strength (*F*[*t*]) in the PFL (Fig. 5b), and with exposure to a sufficient magnitude and duration of elevated matrix elastic modulus (Fig. 5c). Following bifurcation to an activated state, the model predicted that the PFL would render activation irreversible, a phenomenon analogous to “mechanical memory” reported in stem cells^11^.

To validate these predictions experimentally (Fig. 5d), we cultured CFs on a stiff matrix for 7 days, an interval sufficient to upregulate the activation marker protein α-SMA and other activation markers (Col 1, PDGFR-α and TCF21), and then transferred them to a soft hydrogel for further culture for 1 day (St7So1) or 7 days (St7So7). Transfer to soft matrices after 7 days reduced expression of activation markers (Fig. 5e, f), indicating that CFs maintained some memory of the 7-day exposure to stiff matrices, but that this activation was still reversible. Later, we further checked whether mechanical PFL still existed even when CFs were transferred to soft matrix. CFs were cultured on the stiff matrix for 14 days and then transferred to a soft hydrogel for 7 days (St14So7). Interestingly, immunofluorescence analysis showed that the levels of integrin β1 and Piezo1 were also high even when CFs were transferred to the soft matrix (consistent with the control group, St21, cultured on the stiff matrix for 21 days) (Fig. 5g–j). Therefore, we concluded that such persistent high levels of integrin β1 and Piezo1 are caused by the mechanical positive feedback between them.

Tissue elastic modulus plays a key role in cardiac fibrosis^45^, and others have thus attempted to treat fibrosis by reducing tissue elastic modulus (e.g., using LOX inhibitors^46^); however, such treatments have not been successful^47,48^. By modeling the newly identified PFL, our simulations predicted a phase diagram of conditions over which the CF activation should become reversible (Fig. 5k, l and Supplementary Fig. S20). The simulation results (Fig. 5m) show that simultaneously inhibiting the mechanical PFL (by adding the inhibitor of integrin or Piezo1) and decreasing matrix elastic modulus (Line 3) can reverse CF activation to the greatest extent (the same degree as in no memory case, Line 1). Decreased integrin or Piezo1 (Line 4) or matrix elastic modulus (Line 2) alone can only partially reverse cellular memory-mediated CF activation. Later, we investigated the effects of combination of inhibitors at different ratios on CF activation. Simulation results show three regions: (I) irreversible region where no inhibitors of integrin and Piezo1 are added; (II) partially reversible region where only one type of inhibitors is added; (III) reversible region where both inhibitors of integrin and Piezo1 are added. Such simulation results and validation experimentally show that, with decreased matrix elastic modulus, simultaneously inhibiting integrin and Piezo1 are best to reverse CF activation (Supplementary Figs. S21a, S22a, b). Later, we investigated the effects of start time (T1) and treatment time (T2) of inhibitors (Supplementary Fig. S21b) on CF activation. In general, starting treatment as early as possible to prolong treatment time can reverse the activation of CFs better. Interestingly, we found that although we started the treatment as early as possible, it might still cause the reactivation of CFs due to the short treatment time (red arrow).

To evaluate these in silico predictions in vitro, we tested experimentally whether the Piezo1 inhibitor GsMTx4 or the combination of the integrin β1 inhibitors PLX4720 and PF562271 would reduce activation of CFs under conditions for which the phenotypic switch had been irreversible (St14So7). Both treatments decreased the expression of α-SMA, nuclear translocation of YAP, and expression of *CTGF*, while increasing the expression of phosphorylated (inactive) YAP (Fig. 5n, o). Thus, interruption of the integrin β1-Piezo1-mediated mechanical PFL and reduction of ECM stiffness reversed CF activation.

## Discussion

**Fig. 6:**
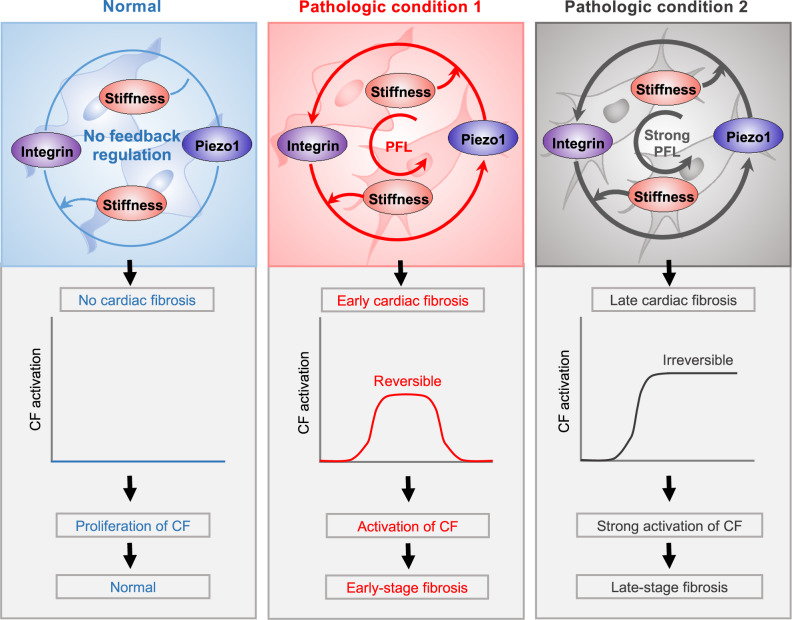
Proposed mechanical PFL, mediated by Piezo1 and integrin β1, that contributes to irreversible CF phenotype. The integrin β1-Piezo1 PFL suggests clues for understanding fibroblast activation during the development of myocardial infarction and fibrotic disease. A bistable switch can be turned on under pathologic conditions by stiffness-mediated activation of the PFL, eventually resulting in irreversible activation of fibroblasts as the disease progresses.

Using integrated in vivo, in vitro, and in silico models, we identified a PFL between Piezo1 and integrin β1 in cardiac fibrosis. The PFL acted as a bistable switch that was shown in vitro to control switching from an irreversible-activated state in CFs during tissue stiffening associated with the progression of cardiac fibrosis. The PFL locked CFs into an irreversible and active state that persists in the absence of high elastic modulus of ECM (Fig. 6). However, by interrupting the PFL through change of matrix elastic modulus and control of integrin β1 and Piezo1, the activation became reversible.

This synergistic role for Piezo1 and integrin β1 adds to their many previously identified individual roles in a range of physiological, developmental, and pathophysiological processes. Piezo1-mediated mechanotransduction is critical for sensory functions in vascular homeostasis, blood pressure and axon growth^49–51^. The fibroblast to myofibroblast transformation is associated with upregulation of several integrins^52–54^. In our experiments, matrix elastic modulus-induced transformation of CF phenotype was associated with integrin β1-mediated maturation of mature focal adhesions (Fig. 3d, g, h) and with increased expression of Piezo1 (Fig. 2c). Neutralization of integrin expression or deletion of integrin β1-reduced α-SMA expression and CF activation, indicating that integrins are indeed critical for transformation of CF phenotype (Fig. 2g). Therefore, exploring the role of integrin and Piezo1 in the development of the disease is very important for developing more effective treatment.

The mathematical model was consistent with two possible pathways for the mechanical PFL. First, mechanical tractions may underlie integrin-mediated activation of Piezo1^39^. Focal adhesion maturation recruits and activates downstream signaling proteins on the FAK-YAP axis^55^ and enhances actin polymerization, stress fiber formation, and mechanical tractions exerted by cells^56^. Second, Piezo1 may promote integrin activation via FAK signaling: a Piezo1-dependent feedforward between glioma mechanotransduction and tissue stiffening can regulate focal adhesion assembly and activate integrin-focal adhesion signaling in gliomas^44^; blood flow perturbations can promote Piezo1-mediated integrin activation and FAK-dependent NF-κB activation in endothelial cells; and Piezo1-depleted endothelial cells show pathologically reduced integrin activation^33,57^.

In our mathematical model, matrix elasticity-mediated PFL is closely relative with irreversible CF activation, and the feedback strength in model is actually a coarse-grained activation rate between piezo1 and integrin. Thus, we can see that there are two parts in ordinary different equations (ODEs) of our model to represent the activation of one species by another molecule: one is elasticity-based linear activation and the other is feedback (specific pathway)-based nonlinear activation.

The mathematical model also provides a potential explanation for cellular phenotype switching in the context of “mechanical memory”, a term referring to how past stimuli affect cellular behaviors, including differentiation or phenotypic switching^58^, long after the stimuli are removed. From this perspective, the PFL bistable switch may work to make reversal of CF activation energetically unfavorable once the duration and magnitude of exposure to stiff matrices are sufficient. The PFL may work in concert with miR-21-mediated mechanical memory^59^, with YAP and miR-21-involved similar responses in fibroblasts to changes in matrix elastic modulus^60,61^.

From the perspective of PFLs, interestingly, this study reveals the first instance in which mechanics has been shown to drive a positive chemical feedback loop. Inhibition of this mechanical PFL in conjunction with reduction of matrix elastic modulus enabled reversal of an otherwise irreversible state of activation. Pharmacological inhibition of the PFL in concert with treatment to reduce collagen synthesis and thus decrease matrix elastic modulus^62,63^ may be an interesting potential molecular therapy. This study demonstrates a role for mechanics in a chemical feedback loop essential to mechanotransduction, and suggests a range of targets for future therapeutic approaches.

## Materials and methods

### Animal experiments

All procedures including purchased male rats (Sprague-Dawley, SD) and experimental protocols followed the Guidelines provided by the Care and Use of Laboratory Animals of Xi’an Jiaotong University. Animals were provided by the Experimental Animal Center of Xi’an Jiaotong University School of Medicine. Before the experiment, all animal facilities were maintained at a temperature of ~23 °C and a humidity of ~40% during the 12-h light–dark cycle. The rats were fed with drinking water and commercial standard diet. The construction of MI model was carried out as described in the previous article^35^. The MI infarction regions of hearts were obtained 7 and 28 days later. Data were collected and analyzed from 21 sites on the MI regions for staining, Western blot and RT-PCR analyses.

### Preparation and characterization of gelatin hydrogels

For preparation of PEG hydrogels, 8-arm PEG maleimide (hexaglycerol) (PEG-MAL, 10 kDa, PengSheng Biolodical) backbone and 8-arm PEG thiol (hexaglycerol) (PEG-SH, 10 kDa, PengSheng Biolodical) crosslinker were used. 8-arm PEG maleimide (PEG-MAL) modified with mono-thiolated peptides was subsequently crosslinked by 8-arm PEG thiol (PEG-SH) via Michael addition^64^. RGD peptides (GCGYGRGDSSPG) (Sangon Biotech, Shanghai) were used for cell adhesion and were covalently conjugated through the Michael addition reaction between the cysteine residues on these peptides and the maleimide on the PEG-MAL backbone. PEG hydrogels modified with 1 mM RGD established integrin adhesions. PEG-MAL (2.5%) and peptides were dissolved in deionized water for 1 h at 37 °C for peptide conjugation. By varying the concentration of PEG-SH used to crosslink the hydrogel (2.5% PEG-MAL as the final concentration) from 1% PEG-SH to 1.625% PEG-SH, the elastic modulus of the PEG hydrogels from 4 kPa (soft), 8 kPa (medium), and 15 kPa (stiff) were altered.

A 200 μm-thick hydrogel was prepared and soaked in phosphate-buffered saline (PBS) overnight to swell to characterize its mechanical properties. After the hydrogels were swelled and balanced, a metal punch was used to prepare a hydrogel disc with a diameter of 8 mm. Then a rotational rheometer (Anton Paar MCR 302, Austria) was used to perform rheological measurements at a temperature of ~25 °C using 8-mm stainless steel parallel plates. When the test was about to start, the normal force should reach ~50 mN by slowly lowering the plate. Finally, the storage modulus (*G*′) and loss modulus (*G*″) of PEG hydrogel were obtained by measurement. The following formula were used to calculate the corresponding stiffnesses^65^:

$${{G}} = \sqrt {{{{G}}}{^\prime} ^2 + {{{G}}}{^{\prime}{^\prime}} ^2}$$

$${{{E}}} = 2{{{G}}}\left( {1 + {{{\nu }}}} \right)$$

where *E* is the elastic modulus of PEG hydrogel to be determined, *ν* represents the material’s Poisson ratio (here, its value is set to 0.5), *G* is the modulus of PEG hydrogel, *G*′ is the storage modulus of PEG hydrogel, and *G*″ is the loss modulus of PEG hydrogel.

The water absorption capacity of PEG hydrogels is determined by the swelling ratio. We measured the swelling weight (*W*_s_) of PEG hydrogel after swelling equilibrium, and then a vacuum freeze dryer (Yaxing Instrument, China) was used to freeze dry and recorded the dry weight (*W*_d_) of the PEG hydrogels after soaking in PBS. The swelling ratio of PEG hydrogels was then calculated as *W*_s_/*W*_d_. The swelling experiments confirmed that the swelling ratio of PEG hydrogels decreased with the increasing concentration of PEG-SH, approaching an unchanged value after the PEG-MAL concentration reached 1.25% (Supplementary Fig. 2b). In combination, we can obtain the PEG hydrogels with different stiffnesses without changing the PEG-MAL concentration.

### Cell isolation and culture

CFs obtained from the hearts of neonatal SD rats (1-day old) were used in this study, as motivated by previous studies^35^. Briefly, neonatal rat myocardium was isolated following euthanasia by cervical dislocation, then cut into pieces and digested into suspended cells using type II collagenase (DIYIBio, China) solution (37 °C, 5 min, seven times). After a 200-mesh was used to filter the mixture, pellets were obtained by centrifuging the mixture. Then, cell culture medium was added to the cell pellets, containing Dulbecco’s modified Eagle’s medium/Ham’s F-12 50/50 mix (DMEM/F-12; CORNING, China), 1% 10 kU/mL penicillin/10 mg/mL streptomycin (Procell, China), and 10% fetal bovine serum (FBS; Gibco, USA). After the mixed cells were cultured in the plate for ~45 min at 37 °C in 5% CO_2_, the supernatant medium (mainly including cardiomyocytes) was discarded and replaced by fresh cell culture medium, and the CFs were isolated from the remaining adherent cells. The CFs were seeded on PEG hydrogels with different stiffness for further experiments.

### Cell transfection and inhibition

CFs were transfected with siRNAs by using Lipofectamine™ 2000 (Thermo Fisher Scientific, USA). The siRNA was used to knockdown Piezo1 in CFs. The targeted sequence of the siRNA directly against rat *Piezo1* RNA was 5′-CGGCCAACAUAAAGAACAUTT. The targeted sequences of siRNAs directly against rat *integrin β1* RNA was 5′-GCCAGAUGGAGUAACAAUATT. The negative control was performed with a scrambled siRNA (5′-UUCUCCGAACGUGUCACGUTT).

CFs were transfected with a plasmid to overexpress YAP. The CFs were seeded on PEG-RGD hydrogel disc with a diameter of 35 mm. Then, a preincubated mixture (including Opti-MEM, DNA, and Lipofectamine™ 2000) was quickly dropped into each culture plate. Approximately 6–8 h after transfection, the medium was removed and replaced with new medium (including FBS and penicillin/streptomycin), and the CFs were allowed to recover for 3 days on the matrices prior to analysis.

For the inhibition treatments, cells were respectively treated with 5 μg/mL integrin β1-blocking antibody (BD Pharmingen™, USA), 2.5 μM GsMTx4 (MedChemExpress, USA), 1 μM PF-562271 (MedChemExpress, USA) and 2 μM PLX-4720 (MedChemExpress, USA), and the control groups were added into the corresponding amount of DMSO diluted in culture media.

### Immunohistochemistry

For heart tissue staining, the obtained tissues (including the normal control (NC), MI-7d and MI-28d heart tissues) were first fixed by 4 % paraformaldehyde and then embedded in paraffin for preservation. After a series of treatments, the samples were blocked with 5% bovine albumin (BSA; MP Biomedicals, USA) for 2 h and washed 3 times with PBS, and then added primary antibodies at 4 °C overnight, containing Piezo1 antibody (1:200; Affinity, DF12083, China), integrin β1 polyclonal antibody (1:1000; Proteintech, 12594-1-AP, China) and α-SMA antibody (1:1000; Cell Signaling Technology, 19245, USA). Secondary antibody (1:100; Dako, P0448, China) was added for 60 min. The current 3,3′-diaminobenzidine tertrahydrochloride (DAB) (Boster, AR1024, China) was used for chromogenic development for 15 s. Cell nuclei were stained with hematoxylin dye (Boster, AR0005, China). The images were captured with an inverted fluorescence microscope (Nikon, Japan) and analyzed by ImageJ 6.0 (National Institutes of Health, USA).

### Immunofluorescence staining and image analysis

For cell staining, cells seeded on PEG-RGD were first fixed with 4% paraformaldehyde (Bioshap) for 10 min, followed by 15 min permeabilization with 0.5% Triton X-100 (Sigma-Aldrich). Then, the samples were blocked with 10% goat serum (Gibco) for 50 min at 37 °C. The primary antibodies used in our study include CD29 (Integrin β1) monoclonal antibody (1:200; Thermo Fisher Scientific, 13-0291-80, USA), α-SMA-FITC antibody (1:400; Sigma, F3777, USA), Piezo1 antibody (1: 100; Affinity, DF12083, China), YAP antibody (Alexa Fluor^®^ 488 Conjugate; 1:100; Cell Signaling Technology, 14729, USA), vinculin antibody (1:250; Abcam, EPR8185, England), paxillin antibody (1:250; Abcam, Y113, England), and Ki-67 (D3B5) antibody (1:400; Cell Signaling Technology, 9129, USA) at 4 °C overnight. Alexa Fluor^®^ 488 (H + L) secondary antibody (1:500; Cell Signaling Technology, A11034, USA) was further incubated at room temperature for 2 h. Cell cytoskeleton was stained by Rhodamine phalloidin (1:500; Invitrogen, R415, USA). DAPI (0.5 μg/mL; Cell Signaling Technology, 4083, USA) was used to stain cell nuclei.

Immunofluorescent staining for YAP/F-actin/DAPI was used to calculate the ratio of nuclear YAP to cytoplasmic YAP (*R*_nc_). Then these areas were determined by using ImageJ. The following formula was used to calculate the YAP n/c ratio, *R*_nc_:

$$R_{\mathrm {{nc}}} = \frac{{I_{{\mathrm {nucleus}}}/A_{{\mathrm {nucleus}}}}}{{(I_{{\mathrm {cell}}} - I_{{\mathrm {nucleus}}})/(A_{{\mathrm {cell}}} - A_{{\mathrm {nucleus}}})}}$$

where *I*_nucleus_ is the total YAP fluorescence intensity of all pixels within the nucleus, as delineated by DAPI staining; *I*_cell_ was that for the entire cell, delineated by F-actin staining; *A*_nucleus_ was the number of pixels in the DAPI-stained nucleus; and *A*_cell_ was the number of pixels in the entire F-actin stained cell.

### Calcium imaging

CFs were incubated with Fluo-4 AM (5 μM; Invitrogen) per the manufacturer’s protocols. The solution containing Hanks’ balanced salt solution (HBSS) with 2 mM Ca^2+^ was used to wash CFs. Imaging was performed at 37 °C in 5% CO_2_ by using an Olympus FV3000 scanning microscope using 20× objective lens. Images were recorded every 3 s over 4 min. Yoda1 resuspended in DMSO at 20 mM was added at a final concentration of 20 µM in HBSS medium. Decreases in fluorescence were achieved by perfusing 2.5 µM GsMTx4.

### Western blot analysis

The acquired tissues and cells were lysed with RIPA on ice for 30 min. Then, proteins were harvested by a low temperature high speed centrifuge for 10 min. The primary antibodies used in our study include Piezo1 antibody (1:1000; Affinity, DF12083, China), GAPDH antibody (1:1000; Cell Signaling Technology, 2118, USA), α-SMA antibody (1:1000; Cell Signaling Technology, 19245, USA), pMAPK antibody (1:1000; Cell Signaling Technology, 9101, USA), CTGF antibody (1:1000; Abcam, ab6992, England), and YAP antibody (1:1000; Cell Signaling Technology, 14074, USA). Goat anti-rabbit HRP-conjugated antibody (1:5000; Cell Signaling Technology, 7074, USA) was used as secondary antibody. Then the ultra-high sensitivity ECL kit (MedChemExpress, HY-K1005, USA) was used for chromogenic development for 2 min. The bands were detected by a chemiluminescence system (ChemiScope 3300 Mini, China). ImageJ program was used to quantify the exposed protein bands.

### mRNA expression analysis

An RNA extraction kit (TaKaRa, China) was used to extract the total RNAs from the CFs seeded on PEG hydrogel substrates. A high capacity RevertAid First Strand cDNA Synthesis Kit (Invitrogen, USA) was used to transcribe the total RNAs into cDNA. RT-PCR was running on a 7500 Fast Real-Time PCR System (Thermo Fisher Scientific, USA) and reacted by using SYBR Green Premix Pro Taq (Accurate Biology, China). Relative gene expression was quantified by using the 2^−ΔΔCt^ method and was internally standardized to glyceraldehyde-3-phosphate dehydrogenase (*GAPDH*). The following primer sequences were used in our study: *Piezo1*: forward 5′-CGGACAGTGAGGAGGAAGAGGAG-3′, reverse 5′-CCTGTTCACGACGCTGCCTTAG-3′; *integrin β1*: forward 5′-AAAATGGACGAAAGTGCTCTAAC-3′, reverse 5′-TGGGACTTGCTGGGATGC-3′; *α-SMA*: forward 5′-CGATAGAACACGGCATCATC-3′, reverse 5′-CATCAGGCAGTTCGTAGCTC-3′; *Col 1*: forward 5′-GGTGAGACAGGCGAACAAGGTG-3′, reverse 5′-GCCAGGAGAGCCAGGAGGAC-3′; *TGF-β1*: forward 5′-CTTCAATACGTCAGACATTCGG-3′, reverse 5′-CACAGTTGACTTGAATCTCTGC-3′; transcription factor 21 (*TCF21*): forward 5′-ACTGGCTCCCTCAGCGATGTAG-3′, reverse 5′-ACCCTCCTCGGTGCTCTCATTG-3′; adipocyte platelet-derived growth factor receptor-α (*PDGFR-α*): forward 5′-CAGGCAGGGCTTCAACGGAAC-3′, reverse 5′-AGTCTGGCGTGTGTCCATCTCC-3′; *YAP*: forward 5′-TACATAAACCATAAGAACAAGACCACA-3′, reverse 5′-GCTTCACTGGAGCACTCTGA-3′; cysteine-rich 61 (*CYR61*): forward 5′-ATCTCCACACGAGTTACCAATGACAAC-3′, reverse 5′-CCACAAGGACGCACTTCACAGATC-3′; *AT*_*1*_*R*: forward 5′-GCTTCAACCTCTACGCCAGTGTG-3′, reverse 5′-CGAGACTTCATTGGGTGGACGATG-3′; *CTGF*: forward 5′-CTCTTCTGCGACTTCGGCTC-3′, reverse 5′-GTACACGGACCCACCGAAGA-3′; *GAPDH*: forward 5′-CTTCTCTTGTGACAAAGTGGACAT-3′, reverse 5′-CTTGCCGTGGGTAGAGTCAT-3′.

### Flow cytometry

CFs were trypsinized and then resuspended in PBS containing 10% GBS to a concentration of 2 × 10^6^ cells/mL. Cells were stained with CD29 (Integrin β1) monoclonal antibody, and analyzed with a flow cytometer (BD Biosciences, USA). Data analysis was performed using Flow Jo analysis software (Tree Star).

### Mathematical model

We developed a mathematical model of the mechanical positive feedback loop between integrin β1 and Piezo1. The model built from that of Yeo et al.^19^. The minimal dynamical model included three classes of molecules: Piezo1, integrin β1, and their downstream targets. The model captured the regulation methods observed experimentally: (1) integrin β1-mediated activation of Piezo1 on substrates of high stiffness; (2) Piezo1-medaited activation of integrin β1 on substrates of high stiffness; and (3) expression of downstream fibrosis-related molecules upregulated by integrin β1 and Piezo1. The dynamics of these chemical reactions were described via Michaelis–Menten kinetics. Here, feedback strength in our simulation means the rate that inactive integrins (or Piezo1) are activated by active Piezo1 (or integrins). We then used a set of coupled ordinary differential equations to simulate mechanical positive feedback loop dynamics. The mathematical equations are as follows:

$$\frac{{{\mathrm {d}}\left[ {C_{\mathrm {I}}} \right]}}{{{\mathrm {d}}t}} = \left[ {k_{1f}S\left( t \right) + F\left( t \right)\frac{{[C_{\mathrm {P}}]^n}}{{1 + [C_{\mathrm {P}}]^n}}} \right]\left( {1 - \left[ {C_{\mathrm {I}}} \right]} \right) - k_{1r}\left[ {C_{\mathrm {I}}} \right]$$

$$\frac{{{\mathrm {d}}\left[ {C_{\mathrm {P}}} \right]}}{{{\mathrm {d}}t}} = \left[ {k_{2f}S\left( t \right) + F\left( t \right)\frac{{[C_{\mathrm {I}}]^n}}{{1 + [C_{\mathrm {I}}]^n}}} \right]\left( {1 - \left[ {C_{\mathrm {P}}} \right]} \right) - k_{2r}\left[ {C_{\mathrm {P}}} \right]$$

$$\frac{{{\mathrm {d}}\left[ {C_{\mathrm {a}}} \right]}}{{{\mathrm {d}}t}} = k_{3f}\left( {\left[ {C_{\mathrm {I}}} \right] + \left[ {C_{\mathrm {P}}} \right]} \right)\left( {1 - \left[ {C_{\mathrm {a}}} \right]} \right) - k_{3r}\left[ {C_{\mathrm {a}}} \right]$$

where *S*(*t*) is the time-dependent substrate stiffness (from 0 to 100); the feedback strength *F*(*t*) was time dependent (from 0 at the initial perturbation of substrate stiffness to 1 after a prescribed time); *k*_1*f*_ (1 s^−1^), *k*_2*f*_ (1 s^−1^) and *k*_3*f*_ (1 s^−1^) represent the basal rate constants of protein production; *k*_1*r*_ (0.1 s^−1^), *k*_2*r*_ (0.1 s^−1^) and *k*_3*r*_ (0.1 s^−1^) are rate constants; *n* is the Hill coefficient which represents the activation threshold; and [*C*_I_], [*C*_P_] and [*C*_a_] represent the concentrations of integrin β1, Piezo1 and downstream fibrotic marker proteins, respectively. The main conclusions drawn from the model were insensitive to the values chosen for rate constants. All concentrations were normalized to 1. Equations were solved numerically using Matlab (The Mathworks, Natick, MA) or GraphPad Prism 8 (GraphPad Software, San Diego, CA, USA).

### Statistical analysis

The GraphPad Prism 8 (GraphPad Software, San Diego, CA, USA) or Microsoft Excel 2017 was used for statistical analyses. Data were performed as the mean ± standard deviation (SD). Two-tailed Student’s *t*-test was used to compare the difference between two samples. One-way analysis of variance (ANOVA) with Tukey post hoc testing and Kruskal–Wallis testing was performed for comparisons between three or four samples (*P < 0.05, **P < 0.01, ***P < 0.001, and ****P < 0.0001).

## Supplementary information

Supplementary Fig S1

Supplementary Fig S18

Supplementary Fig S2

Supplementary Fig S3

Supplementary Fig S4

Supplementary Fig S5

Supplementary Fig S6

Supplementary Fig S7

Supplementary Fig S8

Supplementary Fig S9

Supplementary Fig S10

Supplementary Fig S11

Supplementary Fig S12

Supplementary Fig S13

Supplementary Fig S14

Supplementary Fig S15

Supplementary Fig S16

Supplementary Fig S17

Supplementary Fig S19

Supplementary Fig S20

Supplementary Fig S21

Supplementary Fig S22

## References

[1] Lebar T (2014). A bistable genetic switch based on designable DNA-binding domains. Nat. Commun..

[2] Wunderer J (2019). A mechanism for temporary bioadhesion. Proc. Natl Acad. Sci. USA.

[3] Liu H (2020). Control of fibroblast shape in sequentially formed 3D hybrid hydrogels regulates cellular responses to microenvironmental cues. NPG Asia Mater..

[4] Lahavbaratz S, Sudakin V, Ruderman JV, Hershko A (1995). Reversible phosphorylation controls the activity of cyclosome-associated cyclin-ubiquitin ligase. Proc. Natl Acad. Sci. USA.

[5] Chiarugi P (2003). Reactive oxygen species as essential mediators of cell adhesion: the oxidative inhibition of a FAK tyrosine phosphatase is required for cell adhesion. J. Cell Biol..

[6] Erdmann T, Schwarz US (2006). Bistability of cell–matrix adhesions resulting from nonlinear receptor–ligand dynamics. Biophys. J..

[7] Rognoni L (2014). Force-dependent isomerization kinetics of a highly conserved proline switch modulates the mechanosensing region of filamin. Proc. Natl Acad. Sci. USA.

[8] Cao L (2012). Phage-based molecular probes that discriminate force-induced structural states of fibronectin in vivo. Proc. Natl Acad. Sci. USA.

[9] Becher I (2018). Pervasive protein thermal stability variation during the cell cycle. Cell.

[10] Zhang K (2018). Adaptable hydrogels mediate cofactor-assisted activation of biomarker-responsive drug delivery via positive feedback for enhanced tissue regeneration. Adv. Sci..

[11] Yang C, Tibbitt MW, Basta L, Anseth KS (2014). Mechanical memory and dosing influence stem cell fate. Nat. Mater..

[12] Carlos GA (2022). Coronary microcirculation damage in anthracycline cardiotoxicity. Cardiovac. Res..

[13] Driesen RB (2014). Reversible and irreversible differentiation of cardiac fibroblasts. Cardiovac. Res..

[14] Xiong W, Ferrell JE (2003). A positive-feedback-based bistable ‘memory module’ that governs a cell fate decision. Nature.

[15] Xiong, W. & Ferrell, J. E. Jr. A positive-feedback-based bistable ‘memory module’ that governs a cell fate decision. *Nature* **426**, 460–465 (2003). 10.1038/nature02089 14647386

[16] Ng AH (2019). Modular and tunable biological feedback control using a de novo protein switch. Nature.

[17] Chang DE (2010). Building biological memory by linking positive feedback loops. Proc. Natl Acad. Sci. USA.

[18] Sarkar S (2019). Anticipating critical transitions in pithelial-hybrid-mesenchymal cell-fate determination. Proc. Natl Acad. Sci. USA.

[19] Yeo S-Y (2018). A positive feedback loop bi-stably activates fibroblasts. Nat. Commun..

[20] Meng Z (2018). RAP2 mediates mechanoresponses of the Hippo pathway. Nature.

[21] Swift J, Discher DE (2014). The nuclear lamina is mechano-responsive to ECM elasticity in mature tissue. J. Cell Sci..

[22] Friedland JC, Lee MH, Boettiger D (2009). Mechanically activated integrin switch controls α5β1 function. Science.

[23] Coste B (2010). Piezo1 and Piezo2 are essential components of distinct mechanically activated cation channels. Science.

[24] Kim SE, Coste B, Chadha A, Cook B, Patapoutian A (2012). Piezo in mechanical nociception. Nature.

[25] Ieda M (2009). Cardiac fibroblasts regulate myocardial proliferation through β1 integrin signaling. Dev. Cell.

[26] Burgess ML (1994). Integrin-mediated collagen gel contraction by cardiac fibroblasts-Effects of angiotensin II. Circ. Res..

[27] Blythe NM (2019). Mechanically activated Piezo1 channels of cardiac fibroblasts stimulate p38 mitogen-activated protein kinase activity and interleukin-6 secretion. J. Biol. Chem..

[28] Whasil L (2014). Synergy between Piezo1 and Piezo2 channels confers high-strain mechanosensitivity to articular cartilage. Proc. Natl Acad. Sci. USA.

[29] Murthy SE, Dubin AE, Patapoutian A (2017). Piezos thrive under pressure: mechanically activated ion channels in health and disease. Nat. Rev. Mol. Cell Biol..

[30] van Putten S, Shafieyan Y, Hinz B (2016). Mechanical control of cardiac myofibroblasts. J. Mol. Cell. Cardiol..

[31] Emig R (2021). Piezo1 channels contribute to the regulation of human atrial fibroblast mechanical properties and matrix stiffness sensing. Cells.

[32] Baratchi S (2020). Transcatheter aortic valve implantation represents an anti-inflammatory therapy via reduction of shear stress-induced, piezo-1-mediated monocyte activation. Circulation.

[33] Albarran-Juarez J (2018). Piezo1 and G(q)/G(11) promote endothelial inflammation depending on flow pattern and integrin activation. J. Exp. Med..

[34] Schroer AK, Merryman WD (2015). Mechanobiology of myofibroblast adhesion in fibrotic cardiac disease. J. Cell Sci..

[35] Niu L (2020). Matrix stiffness controls cardiac fibroblast activation through regulating YAP via AT(1)R. J. Cell. Physiol..

[36] Schluter KD, Wollert KC (2004). Synchronization and integration of multiple hypertrophic pathways in the heart. Cardiovasc. Res..

[37] Schnittert J, Bansal R, Storm G, Prakash J (2018). Integrins in wound healing, fibrosis and tumor stroma: high potential targets for therapeutics and drug delivery. Adv. Drug Deliv. Rev..

[38] Cinar E (2015). Piezo1 regulates mechanotransductive release of ATP from human RBCs. Proc. Natl Acad. Sci. USA.

[39] Pathak MM (2014). Stretch-activated ion channel Piezo1 directs lineage choice in human neural stem cells. Proc. Natl Acad. Sci. USA.

[40] Li J (2014). Piezol integration of vascular architecture with physiological force. Nature.

[41] Gudipaty SA (2017). Mechanical stretch triggers rapid epithelial cell division through Piezo1. Nature.

[42] Shimojo N (2015). Tenascin-C may accelerate cardiac fibrosis by activating macrophages via the integrin αVβ3/nuclear factor-κB/interleukin-6 axis. Hypertension.

[43] Dalla Costa AP (2010). FAK mediates the activation of cardiac fibroblasts induced by mechanical stress through regulation of the mTOR complex. Cardiovasc. Res..

[44] Chen X (2018). A feedforward mechanism mediated by mechanosensitive ion channel Piezo1 and tissue mechanics promotes glioma aggression. Neuron.

[45] Conrad CH, Brooks WW, Hayes JA, Sen S, Bing O (1995). Myocardial fibrosis and stiffness with hypertrophy and heart failure in the spontaneously hypertensive rat. Circulation.

[46] Barry-Hamilton V (2010). Allosteric inhibition of lysyl oxidase-like-2 impedes the development of a pathologic microenvironment. Nat. Med..

[47] Denton CP (2007). Recombinant human anti-transforming growth factor beta1 antibody therapy in systemic sclerosis: a multicenter, randomized, placebo-controlled phase I/II trial of CAT-192. Arthritis Rheum..

[48] Stupp R (2014). Cilengitide combined with standard treatment for patients with newly diagnosed glioblastoma with methylated MGMT promoter: a multicentre, randomised, open-label, phase 3 trial. Lancet Oncol..

[49] Ranade SS (2014). Piezo1, a mechanically activated ion channel, is required for vascular development in mice. Proc. Natl Acad. Sci. USA.

[50] Wang, S. et al. Endothelial cation channel PIEZO1 controls blood pressure by mediating flow-induced ATP release. *J. Clin. Invest.* **126**, 4527–4536 (2016). 10.1172/JCI87343 PMC5127677 27797339

[51] Koser DE (2016). Mechanosensing is critical for axon growth in the developing brain. Nat. Neurosci..

[52] Talior-Volodarsky I, Connelly KA, Arora PD, Gullberg D, McCulloch CA (2012). alpha 11 integrin stimulates myofibroblast differentiation in diabetic cardiomyopathy. Cardiovasc. Res..

[53] Babbitt CJ, Shai SY, Harpf AE, Pham CG, Ross RS (2002). Modulation of integrins and integrin signaling molecules in the pressure-loaded murine ventricle. Histochem. Cell Biol..

[54] Chao C, Li R, Ross RS, Manso AM (2016). Integrins and integrin-related proteins in cardiac fibrosis. J. Mol. Cell. Cardiol..

[55] Cheng B (2020). Nanoscale integrin cluster dynamics controls cellular mechanosensing via FAKY397 phosphorylation. Sci. Adv..

[56] Kechagia JZ, Ivaska J, Roca-Cusachs P (2019). Integrins as biomechanical sensors of the microenvironment. Nat. Rev. Mol. Cell Biol..

[57] Petzold T (2009). Focal adhesion kinase modulates activation of NF-κB by flow in endothelial cells. Am. J. Physiol. Cell Physiol..

[58] Ma W, Trusina A, El-Samad H, Lim WA, Chao T (2009). Defining network topologies that can achieve biochemical adaptation. Cell.

[59] Chen XL (2017). MicroRNA-21 preserves the fibrotic mechanical memory of mesenchymal stem cells. Nat. Mater..

[60] Fan D, Creemers EE, Kassiri Z (2014). Matrix as an interstitial transport system. Circ. Res..

[61] Liu F (2015). Mechanosignaling through YAP and TAZ drives fibroblast activation and fibrosis. Am. J. Physiol. Lung Cell. Mol. Physiol..

[62] Yamaguchi Y (2012). A peptide derived from endostatin ameliorates organ fibrosis. Sci. Transl. Med..

[63] Tomcik M (2014). Heat shock protein 90 (Hsp90) inhibition targets canonical TGF-β signalling to prevent fibrosis. Ann. Rheum. Dis..

[64] Zhu H (2018). The relationship between thiol-acrylate photopolymerization kinetics and hydrogel mechanics: an improved model incorporating photobleaching and thiol-Michael addition. J. Mech. l Behav. Biomed. Mater..

[65] Chaudhuri O (2014). Extracellular matrix stiffness and composition jointly regulate the induction of malignant phenotypes in mammary epithelium. Nat. Mater..

