# Supplementary material for: A positive mechanobiological feedback loop controls bistable switching of cardiac fibroblast phenotype
Source: Cell Discov. 2022 Sep 6;8:84. doi: 10.1038/s41421-022-00427-w (PMC9448780; doi:10.1038/s41421-022-00427-w)
Supplement: Supplementary file 1 — Supplementary Fig S1 [file 41421_2022_427_MOESM1_ESM.pdf]

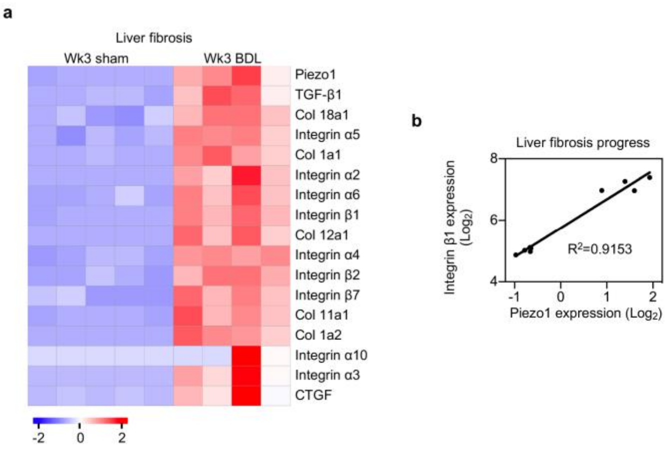

Supplementary Fig. S1 | Heatmap showing different genes expression by cardiac injury. a, Online-accessible GEO data analysis of Piezo1, integrins, collagens and CTGF expression in liver fibrosis (Bile duct ligation (BDL) -mediated liver fibrosis). **b**, Correlation analysis between the expression of Piezo1 and integrin β1 in the liver with sham and fibrosis.
