# Supplementary material for: A positive mechanobiological feedback loop controls bistable switching of cardiac fibroblast phenotype
Source: Cell Discov. 2022 Sep 6;8:84. doi: 10.1038/s41421-022-00427-w (PMC9448780; doi:10.1038/s41421-022-00427-w)
Supplement: Supplementary file 2 — Supplementary Fig S18 [file 41421_2022_427_MOESM2_ESM.pdf]

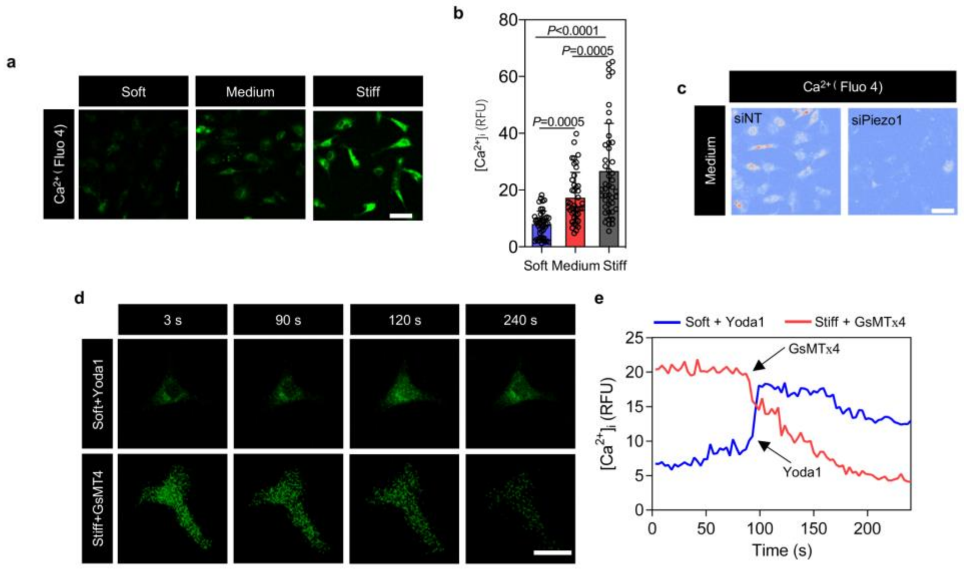

Supplementary Fig. S18 | Piezo1 activity elicits spontaneous Ca^{2+} signals. a, Representative micrographs of CFs challenged with increasing stiffness and analyzed for their responses using Ca^{2+} imaging (Fluo-4 AM). Scale bar, 50 µm. **b**, Quantification of mean fluorescence intensity values (RFU, relative fluorescence units) of CFs perfused with different stiffness (n=42-46 cells). **c**, Ca^{2+} imaging (Fluo-4 AM) analysis when CFs were transfected with siPiezo1 and siNT. Scale bar, 50 µm. **d**, Fluo-4-loaded CFs were exposed to 10 µM Yoda1 on soft matrix and 2.5 µM GsMTx4 on stiff matrix. Scale bar, 10 µm. **e**, Quantification of the mean fluorescence intensity values (RFU, relative fluorescence units) of CFs perfused with 10 µM Yoda1 on soft matrix and 2.5 µM GsMTx4 on stiff matrix. Scale bar, 50 µm.
