# Supplementary material for: A positive mechanobiological feedback loop controls bistable switching of cardiac fibroblast phenotype
Source: Cell Discov. 2022 Sep 6;8:84. doi: 10.1038/s41421-022-00427-w (PMC9448780; doi:10.1038/s41421-022-00427-w)
Supplement: Supplementary file 3 — Supplementary Fig S2 [file 41421_2022_427_MOESM3_ESM.pdf]

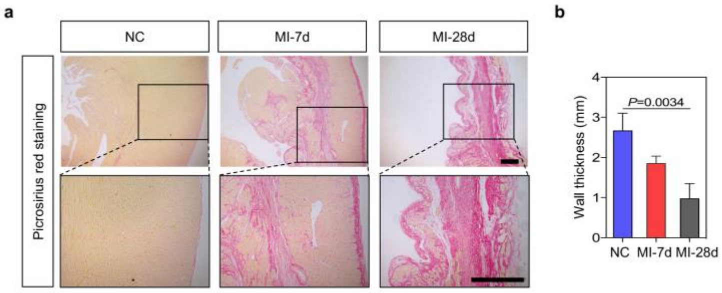

Supplementary Fig. S2 | Characterization of the heart after MI at 7 days and 28 days. a, Representative images of picrosirius red stained sections from NC and MI rats. Scale bar, 500 µm. **b**, Quantification of the MI area wall thickness compared with NC.
