# Supplementary material for: A positive mechanobiological feedback loop controls bistable switching of cardiac fibroblast phenotype
Source: Cell Discov. 2022 Sep 6;8:84. doi: 10.1038/s41421-022-00427-w (PMC9448780; doi:10.1038/s41421-022-00427-w)
Supplement: Supplementary file 4 — Supplementary Fig S3 [file 41421_2022_427_MOESM4_ESM.pdf]

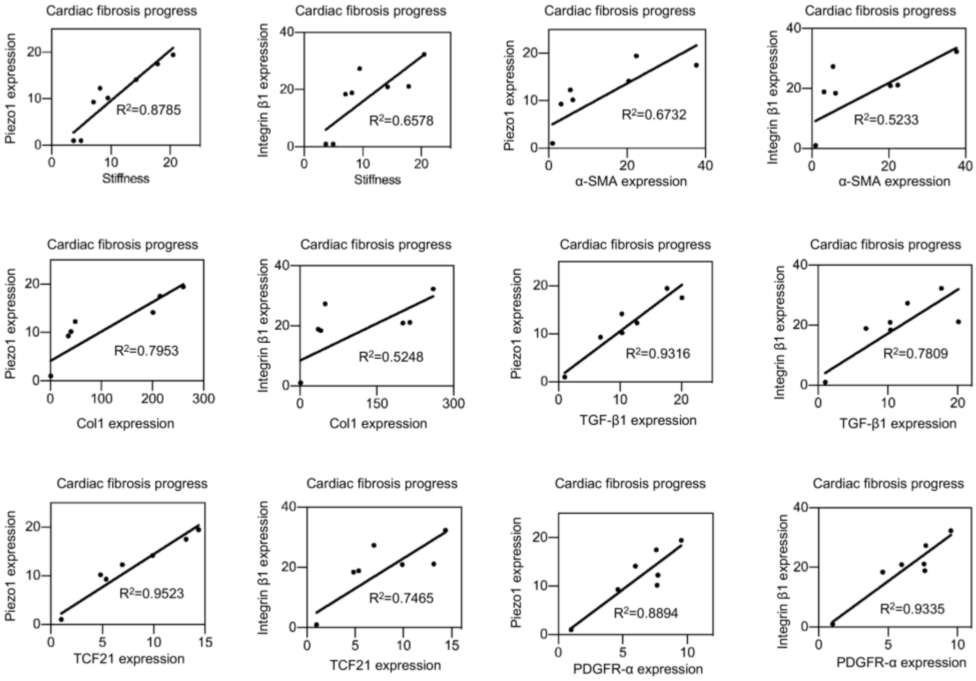

Supplementary Fig. S3 | Correlation analysis between the expression of Piezo1 and integrin β1 *in vivo* and tissue stiffening, and the development of cardiac fibrosis. Correlation analysis between the expression of Piezo1 and integrin with tissue stiffening, and with the development of cardiac fibrosis.
