# Supplementary material for: A positive mechanobiological feedback loop controls bistable switching of cardiac fibroblast phenotype
Source: Cell Discov. 2022 Sep 6;8:84. doi: 10.1038/s41421-022-00427-w (PMC9448780; doi:10.1038/s41421-022-00427-w)
Supplement: Supplementary file 5 — Supplementary Fig S4 [file 41421_2022_427_MOESM5_ESM.pdf]

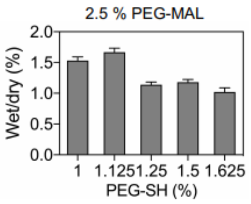

Supplementary Fig. S4 | The characteristic of gelatin hydrogels. Swelling tests of gelatin hydrogels. The concentration of PEG-MAL was 2.5 %wt.
