# Supplementary material for: A positive mechanobiological feedback loop controls bistable switching of cardiac fibroblast phenotype
Source: Cell Discov. 2022 Sep 6;8:84. doi: 10.1038/s41421-022-00427-w (PMC9448780; doi:10.1038/s41421-022-00427-w)
Supplement: Supplementary file 6 — Supplementary Fig S5 [file 41421_2022_427_MOESM6_ESM.pdf]

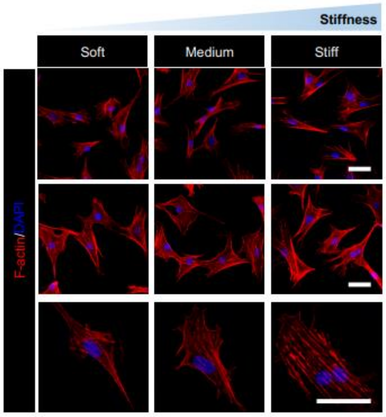

Supplementary Fig. S5 | IF images of CFs in different matrices. F-actin (red) were stained by phalloidin and nucleus (blue) were counter-stained by DAPI. Scale bar, 50 μm.
