# Supplementary material for: A positive mechanobiological feedback loop controls bistable switching of cardiac fibroblast phenotype
Source: Cell Discov. 2022 Sep 6;8:84. doi: 10.1038/s41421-022-00427-w (PMC9448780; doi:10.1038/s41421-022-00427-w)
Supplement: Supplementary file 7 — Supplementary Fig S6 [file 41421_2022_427_MOESM7_ESM.pdf]

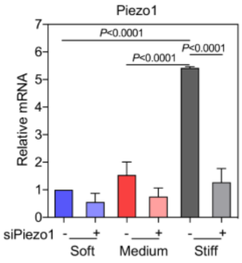

Supplementary Fig. S6 | The verification of Piezo1 knockdown. The relative mRNA levels of YAP determined by RT-PCR when the CFs were transfected with siRNAs targeting Piezo1 after 3 days of culture.
