# Supplementary material for: A positive mechanobiological feedback loop controls bistable switching of cardiac fibroblast phenotype
Source: Cell Discov. 2022 Sep 6;8:84. doi: 10.1038/s41421-022-00427-w (PMC9448780; doi:10.1038/s41421-022-00427-w)
Supplement: Supplementary file 8 — Supplementary Fig S7 [file 41421_2022_427_MOESM8_ESM.pdf]

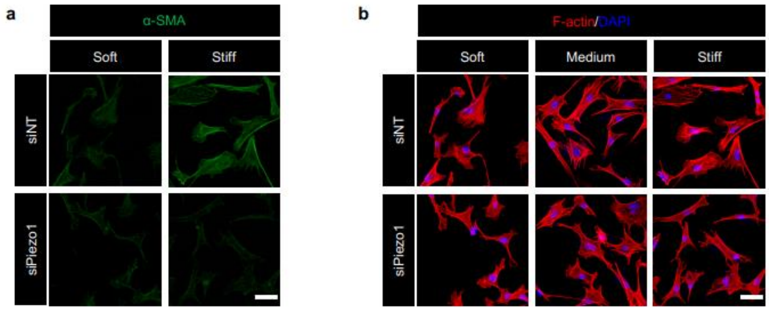

Supplementary Fig. S7 | IF images of Piezo1-depleted CFs in different matrices. **a**, Immunofluorescence analysis indicated the activation of CFs decreased when transfected with siRNAs targeting Piezo1 (green, α-SMA). **b**, F-actin (red) were stained by phalloidin and nucleus (blue) were counter-stained by DAPI. Scale bar, 50 μm.
