# Supplementary material for: A positive mechanobiological feedback loop controls bistable switching of cardiac fibroblast phenotype
Source: Cell Discov. 2022 Sep 6;8:84. doi: 10.1038/s41421-022-00427-w (PMC9448780; doi:10.1038/s41421-022-00427-w)
Supplement: Supplementary file 9 — Supplementary Fig S8 [file 41421_2022_427_MOESM9_ESM.pdf]

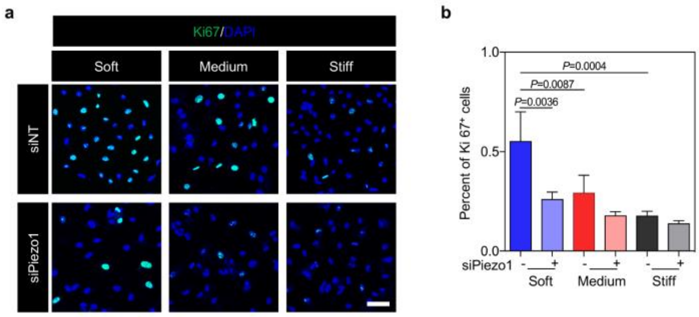

Supplementary Fig. S8 | The proliferation of CFs after Piezo1 knockdown. Immunofluorescence analysis indicated the proliferation of the CFs decreased after transfected with siRNAs targeting Piezo1 (siPiezo1) (blue, nucleus; green, Ki67). b, Quantifications of the percentage of Ki67$^+$ cells (n=27-250 cells).
