# Supplementary material for: A positive mechanobiological feedback loop controls bistable switching of cardiac fibroblast phenotype
Source: Cell Discov. 2022 Sep 6;8:84. doi: 10.1038/s41421-022-00427-w (PMC9448780; doi:10.1038/s41421-022-00427-w)
Supplement: Supplementary file 10 — Supplementary Fig S9 [file 41421_2022_427_MOESM10_ESM.pdf]

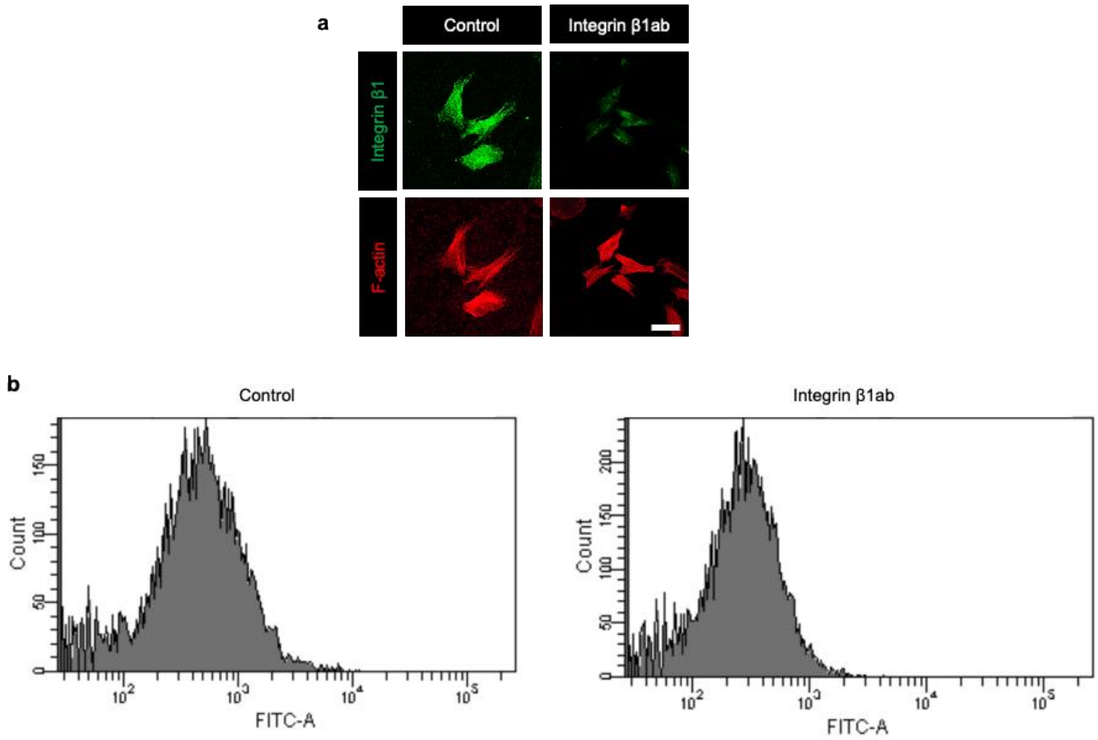

Supplementary Fig. S9 | The verification of antibody-mediated integrin β1 knockdown. a, Immunofluorescence analysis of integrin β1 when CFs were blocked with integrin β1 antibody. Scale bar, 50 μm. **b**, Flow cytometry analysis of RO of integrin β1 antibody.
