# Supplementary material for: A positive mechanobiological feedback loop controls bistable switching of cardiac fibroblast phenotype
Source: Cell Discov. 2022 Sep 6;8:84. doi: 10.1038/s41421-022-00427-w (PMC9448780; doi:10.1038/s41421-022-00427-w)
Supplement: Supplementary file 12 — Supplementary Fig S11 [file 41421_2022_427_MOESM12_ESM.pdf]

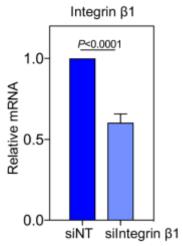


**Supplementary Fig. S11 | The verification of siRNA-mediated integrin β1 knockdown.** RT-PCR analysis of integrin β1 when CFs were transfected with siRNAs targeting integrin β1 (siIntegrin β1) and negative control siRNA (siNT).
