# Supplementary material for: A positive mechanobiological feedback loop controls bistable switching of cardiac fibroblast phenotype
Source: Cell Discov. 2022 Sep 6;8:84. doi: 10.1038/s41421-022-00427-w (PMC9448780; doi:10.1038/s41421-022-00427-w)
Supplement: Supplementary file 13 — Supplementary Fig S12 [file 41421_2022_427_MOESM13_ESM.pdf]

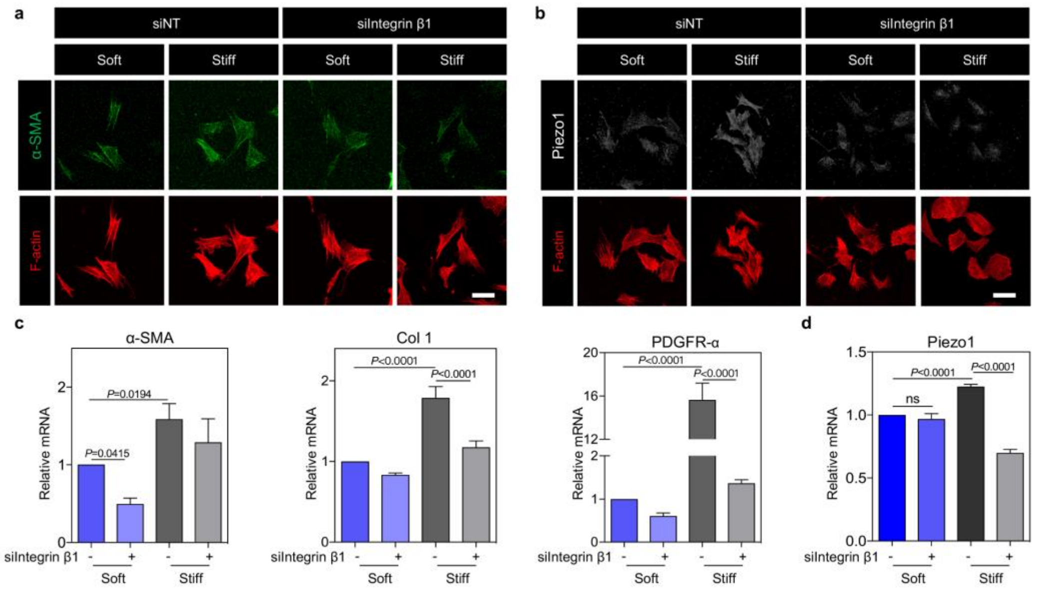

Supplementary Fig. S12 | Integrin promotes activation of CF and enhances the level of Piezo1. a, Immunofluorescence analysis of α-SMA indicated that activation of CFs decreased by using siRNAs silencing of integrin β1 (green, α-SMA; red, F-actin). Scale bar, 50 μm. **b**, Immunofluorescence analysis of Piezo1 indicated that expression of Piezo1 on stiff hydrogels decreased by using siRNAs silencing of integrin β1 (grey, Piezo1; red, F-actin). Scale bar, 50 μm. **c**, RT-PCR analysis of α-SMA, Col 1 and PDGFR-α indicated that activation of CFs decreased following siRNAs silencing of integrin β1. **d**, RT-PCR analysis of Piezo1 indicated that expression of Piezo1 on stiff hydrogels decreased following siRNAs silencing of integrin β1.
