# Supplementary material for: A positive mechanobiological feedback loop controls bistable switching of cardiac fibroblast phenotype
Source: Cell Discov. 2022 Sep 6;8:84. doi: 10.1038/s41421-022-00427-w (PMC9448780; doi:10.1038/s41421-022-00427-w)
Supplement: Supplementary file 14 — Supplementary Fig S13 [file 41421_2022_427_MOESM14_ESM.pdf]

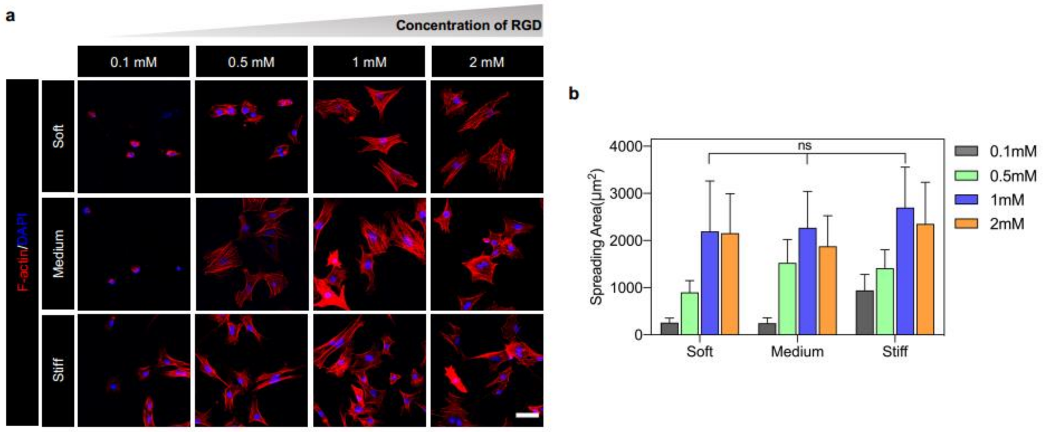

Supplementary Fig. S13 | IF images of CFs in different matrices with the different concentration of RGD. a, Immunofluorescence staining of cells in hydrogels with the different concentration of RGD (blue, nucleus; red, F-actin). Scale bar, 50 μm. **b**, Quantification of the spreading area of cells in hydrogels with the different concentration of RGD (n=17-55 cells).
