# Supplementary material for: A positive mechanobiological feedback loop controls bistable switching of cardiac fibroblast phenotype
Source: Cell Discov. 2022 Sep 6;8:84. doi: 10.1038/s41421-022-00427-w (PMC9448780; doi:10.1038/s41421-022-00427-w)
Supplement: Supplementary file 15 — Supplementary Fig S14 [file 41421_2022_427_MOESM15_ESM.pdf]

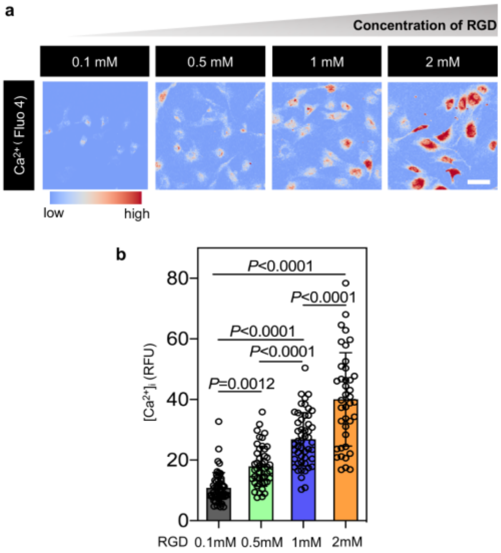

Supplementary Fig. S14 | IF images of CFs in different matrices with the different concentration of RGD. a, Ca^{2+} imaging (Fluo-4 AM) analysis when CFs were cultured in stiff with the increase concentration of RGD. Scale bar, 50 μm. **b**, Quantification of mean fluorescence intensity values (RFU, relative fluorescence units) when CFs were cultured in stiff with the increase concentration of RGD (n≥40 cells).
