# Supplementary material for: A positive mechanobiological feedback loop controls bistable switching of cardiac fibroblast phenotype
Source: Cell Discov. 2022 Sep 6;8:84. doi: 10.1038/s41421-022-00427-w (PMC9448780; doi:10.1038/s41421-022-00427-w)
Supplement: Supplementary file 16 — Supplementary Fig S15 [file 41421_2022_427_MOESM16_ESM.pdf]

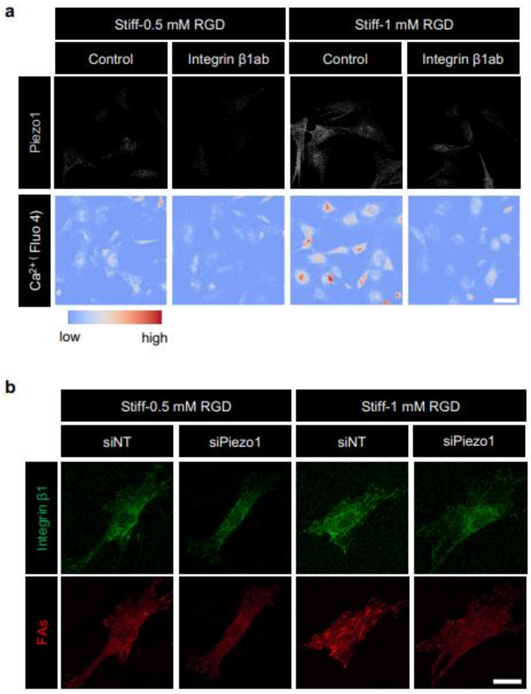

Supplementary Fig. S15 | IF images of CFs in stiff with the different concentration of RGD. a, Immunofluorescence analysis indicated Piezo1 decrease with Integrin β1 antibody treatment in stiff (grey, Piezo1); Ca^{2+} imaging (Fluo-4 AM) analysis when CFs were treated with Integrin β1 antibody treatment in stiff. Scale bar, 50 µm. **b**, Immunofluorescence analysis indicated Integrin β1 decrease and less focal adhesions with siPiezo1 treatment in stiff (green, Integrin β1; red, FAs (Vinculin & Paxllin)). Scale bar, 10 µm.
