# Supplementary material for: A positive mechanobiological feedback loop controls bistable switching of cardiac fibroblast phenotype
Source: Cell Discov. 2022 Sep 6;8:84. doi: 10.1038/s41421-022-00427-w (PMC9448780; doi:10.1038/s41421-022-00427-w)
Supplement: Supplementary file 17 — Supplementary Fig S16 [file 41421_2022_427_MOESM17_ESM.pdf]

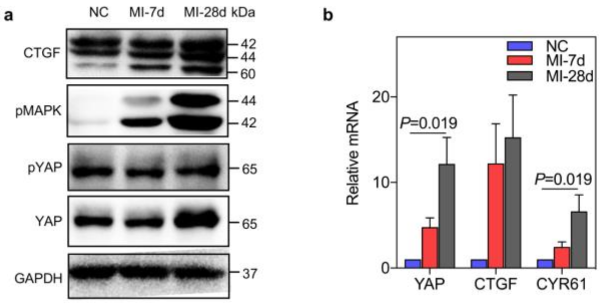

Supplementary Fig. S16 | *In vivo* expression of YAP and its transcriptional targets in the model of cardiac fibrosis. a, Relative protein levels of YAP, pYAP, CTGF and pMAPK determined by Western blot analysis. **b**, RT-PCR analysis of YAP, CTGF and CYR61 in NC and MI rats.
