# Supplementary material for: A positive mechanobiological feedback loop controls bistable switching of cardiac fibroblast phenotype
Source: Cell Discov. 2022 Sep 6;8:84. doi: 10.1038/s41421-022-00427-w (PMC9448780; doi:10.1038/s41421-022-00427-w)
Supplement: Supplementary file 18 — Supplementary Fig S17 [file 41421_2022_427_MOESM18_ESM.pdf]

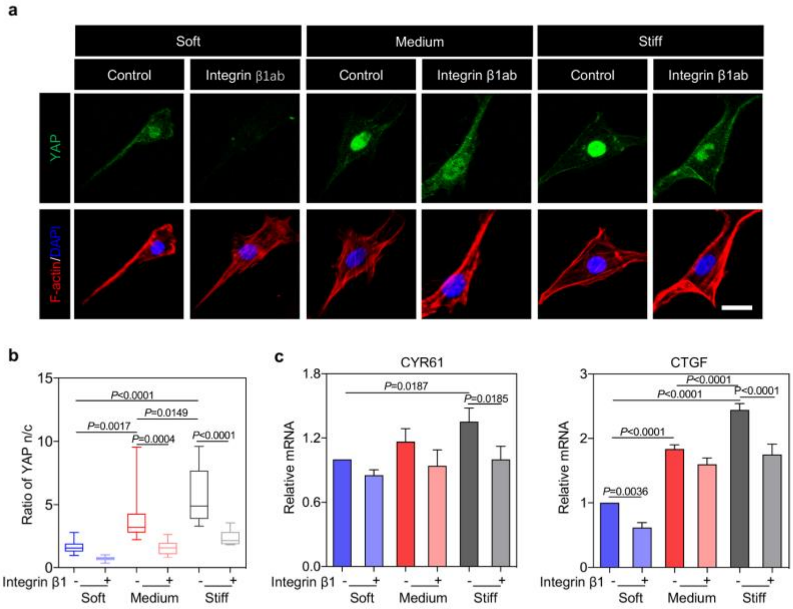

Supplementary Fig. S17 | Integrin was upstream of YAP to govern CFs activation. a, Immunofluorescence analysis indicated YAP in nuclear decrease with integrin β1 blocking antibody (blue, nucleus; green, YAP; red, F-actin). Scale bar, 10 μm. **b**, Quantification of the ratio of nuclear YAP to cytoplasmic YAP and the expression of total YAP for cells (n≥9 cells). **c**, RT-PCR analysis of downstream YAP genetic targets (CYR61 and CTGF) in the CFs with or without integrin blocking antibody.
