# Supplementary figures and images for: A positive mechanobiological feedback loop controls bistable switching of cardiac fibroblast phenotype
Source: Cell Discov. 2022 Sep 6;8:84. doi: 10.1038/s41421-022-00427-w (PMC9448780; doi:10.1038/s41421-022-00427-w)

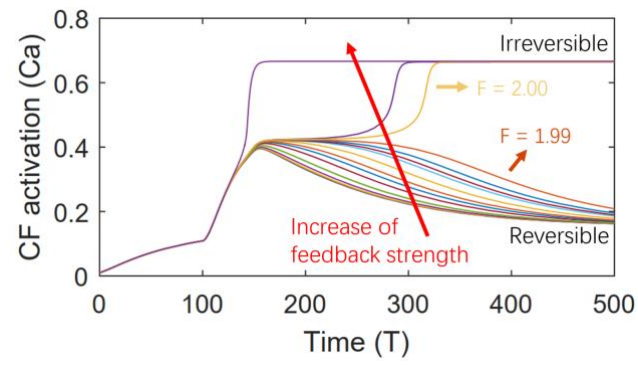

**Supplementary Fig. S20 | The irreversible threshold of feedback strength.**

Supplement: Supplementary file 20 — Supplementary Fig S20 [file 41421_2022_427_MOESM20_ESM.pdf]
