# Supplementary material for: A positive mechanobiological feedback loop controls bistable switching of cardiac fibroblast phenotype
Source: Cell Discov. 2022 Sep 6;8:84. doi: 10.1038/s41421-022-00427-w (PMC9448780; doi:10.1038/s41421-022-00427-w)
Supplement: Supplementary file 21 — Supplementary Fig S21 [file 41421_2022_427_MOESM21_ESM.pdf]

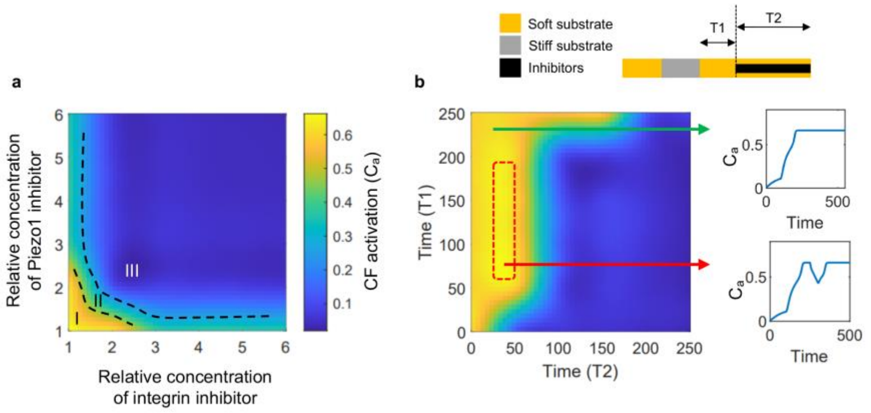

Supplementary Fig. S21 | The effects of combination of inhibitors at different ratio (a) or treatment time (b) on CF activation.
