# Supplementary material for: A positive mechanobiological feedback loop controls bistable switching of cardiac fibroblast phenotype
Source: Cell Discov. 2022 Sep 6;8:84. doi: 10.1038/s41421-022-00427-w (PMC9448780; doi:10.1038/s41421-022-00427-w)
Supplement: Supplementary file 22 — Supplementary Fig S22 [file 41421_2022_427_MOESM22_ESM.pdf]

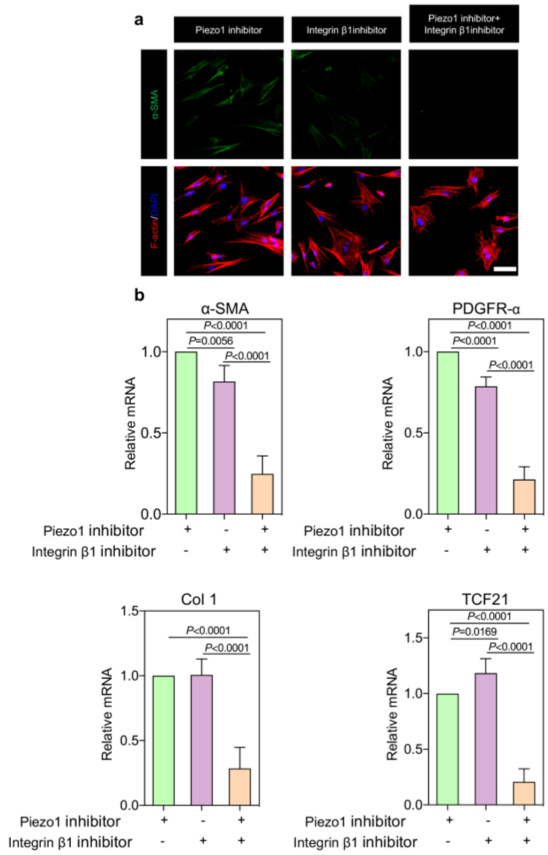

Supplementary Fig. S22 | Validation of the synergistic role between Piezo 1 and integrin β1. a-b, Piezo1 inhibitor, integrin β1 inhibitor and simultaneous blockade reduced activation of fibroblasts in conditions for which phenotypic reversal was otherwise impossible (St14), as seen by Immunofluorescence analysis and RT-PCR (n=6). Scale bar, 50 μm.
